# Characterization of the bZIP Transcription Factor Family in Pepper (*Capsicum annuum* L.): *CabZIP25* Positively Modulates the Salt Tolerance

**DOI:** 10.3389/fpls.2020.00139

**Published:** 2020-02-26

**Authors:** Wen-Xian Gai, Xiao Ma, Yi-Ming Qiao, Bu-Hang Shi, Saeed ul Haq, Quan-Hui Li, Ai-Min Wei, Ke-Ke Liu, Zhen-Hui Gong

**Affiliations:** ^1^ College of Horticulture, Northwest A&F University, Yangling, Shannxi, China; ^2^ Qinghai Academy of Agricultural and Forestry Sciences, Xining, Qinghai, China; ^3^ Tianjin Vegetable Research Center, Tianjin Academy of Agricultural Sciences, Tianjin, China; ^4^ College of Horticulture, Henan Agricultural University, Zhengzhou, Henan, China

**Keywords:** pepper (*Capsicum annuum* L.), Pepper Genome Databases, CabZIP family, CabZIP25, abiotic stress, salt tolerance

## Abstract

The basic leucine zipper (bZIP) proteins compose a family of transcription factors (TFs), which play a crucial role in plant growth, development, and abiotic and biotic stress responses. However, no comprehensive analysis of bZIP family has been reported in pepper (*Capsicum annuum* L.). In this study, we identified and characterized 60 bZIP TF-encoding genes from two pepper genomes. These genes were divided into 10 groups based on their phylogenetic relationships with *bZIP* genes from *Arabidopsis*. Six introns/exons structural patterns within the basic and hinge regions and the conserved motifs were identified among all the pepper bZIP proteins, on the basis of which, we classify them into different subfamilies. Based on the transcriptomic data of Zunla-1 genome, expression analyses of 59 pepper *bZIP* genes (not including *CabZIP25* of CM334 genome), indicated that the pepper *bZIP* genes were differentially expressed in the pepper tissues and developmental stages, and many of the pepper *bZIP* genes might be involved in responses to various abiotic stresses and phytohormones. Further, gene expression analysis, using quantitative real-time PCR (qRT-PCR), showed that the *CabZIP25* gene was expressed at relatively higher levels in vegetative tissues, and was strongly induced by abiotic stresses and phytohormones. In comparing with wild type *Arabidopsis*, germination rate, fresh weight, chlorophyll content, and root lengths increased in the *CabZIP25-*overexpressing *Arabidopsis* under salt stress. Additionally, *CabZIP25-*silenced pepper showed lower chlorophyll content than the control plants under salt stress. These results suggested that *CabZIP25* improved salt tolerance in plants. Taken together, our results provide new opportunities for the functional characterization of bZIP TFs in pepper.

## Introduction

Plant growth, development, and agricultural production are seriously limited by adverse environmental conditions such as drought, salinity, and extreme temperatures. Unfavorable factors cause a series of damages to the plant morphology, physiology, and biochemistry and may significantly cause extensive losses to agricultural production and economic yield (Allakhverdiev et al., 2008). In response, the terrestrial plants began to evolve numerous sophisticated adaptations for survival and productivity challenged by global climate changed 400 million years ago (Scharf et al., 2012). A number of defense mechanisms have been developed through resistance genes, encoding regulatory proteins such as protein kinases, phosphatases, and transcription factors (TFs). TFs are critical components of signaling regulatory networks in response to growth and environmental stresses through recognizing and binding to the specific *cis*-elements in the promoters of the target genes to regulate their expression. Sixty-four TF families have been identified in plant kingdoms, which are classified based on their special DNA binding domains (Pérez-Rodríguez et al., 2010). The basic leucine (Leu) zipper (bZIP) family is one of the largest and most diverse groups among the TF families. In plants, bZIP proteins play important roles in growth and development, besides responding to various biotic/abiotic stresses (Sornaraj et al., 2016).

The bZIP TFs are named according to a highly conserved bZIP domain which is composed of two structural features located on a contiguous α-helix. First, a basic region of around 18 amino acid residues, followed by an invariant N-x7-R/K-x9 motif for nuclear localization and sequence-specific DNA binding. Second, a Leu zipper, consisting of several heptad repeats of Leu or other bulky hydrophobic amino acids (e.g. isoleucine, valine, phenylanine, tryptophan, or methionine), positioned exactly nine amino acids toward the C-terminus, creating an amphipathic helix (Jakoby et al., 2002; Nijhawan et al., 2007). In addition to the bZIP domain, the bZIP TFs also have some other domains, acting as transcriptional activators, such as glutamine-rich motif and phosphorylation site [R/KxxS/T (Furihata et al., 2006; Liao et al., 2008)]. To bind to DNA, the N-terminal half of the basic region binds to the major groove of double-stranded DNA, whereas the C-terminal half of the Leu zipper mediates dimerization to create a superimposed coiled structure [the so-called zipper (Landschulz et al., 1988; Ellenberger et al., 1992)]. The basic region differently binds to DNA sequences with an ACGT core, in particular, the G-box (CACGTG), C-box (GACGTC), and A-box [TACGTA (Izawa et al., 1993; Foster et al., 1994)], but there are also some examples of non-palindromic binding sites (Fukazawa et al., 2000; Liao et al., 2008).

With the development of molecular biology, comprehensive and phylogenetic annotations for the bZIP TF family have been identified and predicted at the genome level in several plant genomes. The bZIP TF family usually contains dozens of members among the examined plants so far. Seventy-five *bZIP* genes have been found in [*Arabidopsis* *Arabidopsis thaliana* (Jakoby et al., 2002)], 55 in tomato [*Solanum lycopersicum* (Li et al., 2015)], 89 in rice [*Oryza sativa* (Nijhawan et al., 2007)], 114 in apple [*Malus domestica* (Li et al., 2016)], 49 in castor bean [*Ricinus communis* (Jin et al., 2014)], and 125 in maize [*Zea mays* (Wei et al., 2012)]. However, only a small proportion of bZIP TFs function has been identified in plants. Previous studies reported that bZIP proteins participated in the differentiation and development of many organs and tissues, such as seed maturation and germination, embryogenesis, flower development, and vascular development in plants (Beachy et al., 1997; Jakoby et al., 2002; Schutze et al., 2008; Shiota et al., 2008; Guan et al., 2009; Toh et al., 2014; Sornaraj et al., 2016). In addition, the bZIP TFs also played important roles in response to signaling pathways and abiotic/biotic stresses, including ABA signaling, light signaling, osmotic, hypoxia, drought, high salinity, cold, and pathogen infections (Rook et al., 1998; Uno et al., 2000; Shimizu et al., 2005; Liao et al., 2008; Schutze et al., 2008; Hsieh et al., 2010; Zander et al., 2012; Alves et al., 2013; Gatz, 2013; E et al., 2014). In *Arabidopsis*, *AtbZIP36* (*ABF2/AREB1*), *AtbZIP37* (*ABF3*), and *AtbZIP38* (*ABF4/AREB2*) genes were involved in response to the ABA signaling, dehydration, and salinity (Uno et al., 2000), in addition, AtbZIP26 (OBF5/TGA5) strongly interacted with NIM1/NPR1 and conferred SAR-independent resistance to *Peronospora parasitica* (Kim and Delaney, 2002). In tomato, SlbZIP33 (SlAREB1), an ABA-regulated bZIP TF, was involved in stress-induced response and acted as key components in regulating the expression of major metabolic pathway-related genes and affected metabolic programming during the fruit ripening (Orellana et al., 2010; Bastias et al., 2014). In rice, the japonica version of bZIP73 (bZIP73^Jap^) conferred cold stress tolerance at both the seedling and reproductive stages through the bZIP71-bZIP73^Jap^-qLTG3-1^Nip^-sugar transport pathway (Liu et al., 2018; Liu et al., 2019).

Pepper (*Capsicum annuum* L.), an important crop, has a wide range of uses, such as vegetable, medicines, spices in cuisines, dyeing agents and so on (Guo et al., 2014; Kim et al., 2014). Currently, only a few of the bZIP TFs are known to regard the biological functions and regulation mechanisms in pepper. Transcripts of some *bZIP* genes such as *CAbZIP1*, *CaAIBZ1*, *CaDILZ1*, and *CaBZ1/PPI1* were shown to be significantly induced by drought and salt stresses (Lee et al., 2002; Hwang et al., 2005; Lee S. C. et al., 2006; Moon et al., 2015; Joo et al., 2018; Lim et al., 2018). Moreover, several *bZIP* genes have also been reported to be involved in signaling pathways and biotic stimuli, including ABA, salicylic acid (SA), methyl jasmonic acid (JA), ethylene, and against pathogen defense (Lee et al., 2002; Lee B. J. et al., 2006; Lee S. C. et al., 2006; Lim et al., 2015; Joo et al., 2018; Lim et al., 2018). The most-studied pepper bZIP TF was CaBZ1/PPI1, which showed specific functional roles in response to abiotic/biotic stresses. In addition, over-expressing *CaBZ1/PPI1* in transgenic potato (*Solanum tuberosum*) increased the expression of ABA and stress-related genes, and enhanced drought tolerance without decreasing the tubers yield (Lee et al., 2002; Hwang et al., 2005; Moon et al., 2015). Pepper bZIP TF, CaAIBZ1, interacting with and degraded by CaASRF1 (*C. annuum* ABA-Sensitive RING Finger E3 ligase 1), positively modulated ABA signaling and the drought stress (Joo et al., 2018). Additionally, *CabZIP63* was up-regulated by *Ralstonia solanacearum* inoculation at high temperature-high humidity and acted as a positive regulator in this process by regulating the expression of *CaWRKY40* (Shen et al., 2016). Collectively, the bZIP TFs family in pepper is an important gene family involved in stress response along with a crucial role in growth and development. However, no systematic studies have been reported on the molecular basis and regulatory mechanisms of pepper bZIP TFs. Thus, we studied and characterized the *CabZIP* genes to explore the underlying complex molecular mechanisms in pepper.

The pepper reference genomes provided an opportunity to identify the gene families and the genome databases facilitated the establishment of expression patterns (Kim et al., 2014; Qin et al., 2014; Liu et al., 2017). In the present study, a total of 60 pepper *bZIP* genes were identified from two pepper genomes. We presented the phylogenetic tree analysis, gene structure analysis, and protein domain organization. Furthermore, the comprehensive expression patterns of *CabZIP* genes were investigated in various tissues and responses to abiotic stresses and signaling pathways using the publicly available transcriptomic data. We also have characterized the function of *CabZIP25* in response to salt stress. Our study provides an important foundation to further reveal the biological functions of the CabZIP TFs family in pepper.

## Materials and Methods

### Genome-Wide Identification of *CabZIP* Genes in Pepper

To identify all the bZIP family members in the pepper genome, both the Hidden Markov Model (HMM) at (http://pfam.xfam.org/) and BLASTP searches were performed. For the HMM search, the HMM models of bZIP (Pfam: PF00170, PF07716) were used as queries to search the pepper Zunla-1 and CM334 proteomes downloaded from the Pepper Genome Platform (PGP) at (http://peppergenome.snu.ac.kr/) (Kim et al., 2014; Qin et al., 2014) and the *E*-value threshold was set at 1. For BLASTP, the published bZIP proteins from *Arabidopsis* (Jakoby et al., 2002) and tomato (Li et al., 2015) were used as query sequences to search against the Zunla-1 and CM334 proteomes in the PGP. All obtained pepper bZIP protein sequences with an *E*-value 1 were assembled to remove redundancy and then subjected to ExPASy (https://prosite.expasy.org/) and SMART (http://smart.embl-heidelberg.de/) to confirm the presence of the bZIP domain. All the putative bZIP protein sequences from CM334 and Zunla-1 genome were aligned using DNAMAN (Lynnon Biosoft, QC, Canada) to manually check and remove the repeated and incomplete sequences. All the confident and non-redundant genes were assigned as pepper *bZIP* genes (*CabZIPs*) and the nomenclature was based on the exact top-down locations of the *bZIP* genes on the Zunla-1 chromosomes 1 to 12 (Jakoby et al., 2002; Jin et al., 2014; Li et al., 2015). The protein sequences of putative CabZIPs were analyzed using ExPASy Compute *P*I/MW tool (https://web.expasy.org/compute_pi/) to obtain the theoretical isoelectric point (*P*I) and molecular weight (MW).

### Phylogenetic Tree Analysis

Multiple sequence alignments were performed by the MUSCLE program in the MEGA-X package with the default parameters (http://www.megasoftware.net/). Twenty AtbZIP proteins (subgroup A, AtbZIP36 and 38; subgroup B, AtbZIP28 and 49; subgroup C, AtbZIP9 and 10; subgroup D, AtbZIP20 and 22; subgroup E, AtbZIP34 and 61; subgroup F, AtbZIP19 and 24; subgroup G, AtbZIP41 and 55; subgroup H, AtbZIP56 and 64; subgroup I, AtbZIP51 and 59; subgroup S, AtbZIP2 and 11) were selected from the ten different subgroups of AtbZIP proteins as references to categorize the CabZIP proteins. The Neighbor-Joining (NJ) phylogenetic tree was constructed using MEGA-X with the bootstrap test calculated using 1,000 iterations, Poisson model and pairwise deletion gaps. Then, the tree was displayed and embellished using the interactive tree of life (https://itol.embl.de/).

### Identification of Conserved Motifs

The conserved motifs of the 60 CabZIP protein sequences were identified using the Multiple Em for Motif Elicitation (MEME version 5.0.5, http://meme-suite.org/tools/meme). The analysis was performed with the following parameters: number of repetitions-any, the maximum number of motifs-10, the optimum motif widths set from 6 to 200 amino acid residues. The conserved motifs were annotated with the InterProScan (http://www.ebi.ac.uk/interpro/).

### Analysis of Gene Structure and Conserved Intron Splicing Site

Both the *CabZIP* cDNA sequences and their corresponding genomic DNA sequences were downloaded from Pepper Hub (http://pepperhub.hzau.edu.cn/), based on the gene IDs and chromosomal location (Liu et al., 2017). All the cDNA and DNA sequences were analyzed using the gene structure display server v2.0 (http://gsds.cbi.pku.edu.cn/), to illustrate the exons/introns organization of the *CabZIP* genes. The particular information on the intron distribution pattern and intron splicing phase within the basic and hinge regions of the bZIP domains were further predicted using Spidey (https://www.ncbi.nlm.nih.gov/sutils/splign/splign.cgi).

### Transcriptomic Data Analysis

To gain an insight into the spatial and temporal expression profile of *CabZIP* genes, during the development and stress responses, transcriptomic data was analyzed (**Supplementary Table S5**). The transcriptome data of 59 *CabZIPs* genes (not including *CabZIP25*) from the Zunla-1 genome, were obtained using the Pepper Hub (http://pepperhub.hzau.edu.cn/) (Kim et al., 2014). The data set contained several pepper tissues/organs at different developmental stages, including leaves (L1-9), flowers (F1-9), fruits (FST0), pericarps (G1-11), placenta (T3-11), and seeds (S3-11). Expression levels of *CabZIP* genes in the leaves/roots during different stresses or hormones treatments were also provided in this profile, such as cold (FL1-6/FR1-6), heat (HL1-6/HR1-6), salt (NaCl; NL1-6/NR1-6), osmotic (mannitol; ML1-6/MR1-6), ABA (AL1-6/AR1-6), gibberellic acid (GA3; G1-6L/GR1-6), indole acetic acid (IAA; IL1-6/IR1-6), JA (JL1-6/JR1-6), and SA (SL1-6/SR1-6). Two separate projects were created, one for the developmental tissues that included data from six organs (leaf, flower, fruit, pericarp, placenta, and seed) and the other for four stress treatments including NaCl, mannitol, heat, and cold stress, and five phytohormone treatments including IAA, GA3, ABA, SA, and JA. The heat maps and hierarchical clustering of the gene expression patterns were performed using the HemI software (http://hemi.biocuckoo.org/down.php) (Deng et al., 2014).

### Plant Materials and Growth Conditions

A breeding pepper line R9 (introduced from the World-Asia vegetable research and development center, PP0042-51) and wild-type (WT) *A. thaliana* plants (ecotype Columbia) were used in this study. The seedlings were grown in a climate-controlled chamber under a 16 h day/8 h night regime and 60 to 70% relative humidity. The temperature was set to 25/18°C day/night for pepper and 22/18°C for *Arabidopsis*.

### Gene Expression Analysis of *CabZIP25* in Pepper

For tissue-specific expression analysis of *CabZIP25* in pepper, seedlings were prepared in a natural glasshouse with the daily temperature of 25 to 29°C and night temperature 16 to 20°C. The samples of roots, stems, leaves, flower buds, fruits, and seeds were collected for expression analysis of different tissues. For expression analysis under phytohormone and stress treatments in pepper, various stress treatments were applied on seedlings of 6-8 true leaves stage and all samples for RNA extraction were collected from young leaves. For heat stress treatment, the seedlings were transferred into a growth chamber at 45°C and leaf tissues were collected at 0, 0.5, 1, 2, 4, 12, and 24 h post treatment, respectively. Salt stress was applied by adding NaCl to a final concentration of 0, 50, 100, 200, 400, and 600 mM in the nutrient solution, respectively, and leaves sample were collected at 6 h post-treatments. Similarly, pepper seedlings were sprayed evenly with 0, 1, 10, 50, 100, and 500µM ABA or JA solution for phytohormone treatments and leaves were sampled at 6 h after treatments. All the samples were collected in quadruplicates, frozen in liquid nitrogen and stored at −80°C prior to the extraction of RNA.

### Salt Resistance Analysis of *CabZIP25* in Transgenic *Arabidopsis*

The full-length CDS of *CabZIP25* was amplified from the line R9 using the *CabZIP25* CDS primers (**Supplementary Table S6**), and the amplification product was cloned into the Xba I and Kpn I site of pVBG2307 vector to create pVBG2307-*CabZIP25* under the control of the CaMV35S promoter. The obtained over-expression vector was transformed into *Agrobacterium tumefaciens* strain GV3101 and was subsequently infiltrated into WT *Arabidopsis* plants (Clough and Bent, 1998). The seeds were screened on Murashige and Skoog (MS) selection medium with kanamycin (50 mg/L) and T3 generation lines OE1, OE2, and OE5 were used for experimental analysis. For salt tolerance evaluation of over-expressing *CabZIP25*, seeds of transgenic and WT *Arabidopsis* were germinated on MS medium supplemented with 0, 100, and 150 mM NaCl. The germination rate was observed every day, and the fresh weight and chlorophyll content were measured at 10-day-old *Arabidopsis* seedlings. The chlorophyll content was measured with the method described previously (Cai et al., 2017). To assess the effect of *CabZIP25* overexpression on *Arabidopsis* roots development, seeds of *CabZIP25*-OE and WT lines were germinated on MS petri dishes containing 0, 100, and 150 mM NaCl. The dishes were placed vertically and the root lengths of transgenic and WT plants were measured after 10 days. Three replicates were performed in this experiment. Data were reported as means ± SD.

### Silencing of *CabZIP25* through Virus-Induced Gene Silencing (VIGS) in Pepper and Salt Treatment for VIGS Seedlings

*CabZIP25*-silenced pepper plants were generated using tobacco rattle virus-based VIGS (Liu et al., 2002). Brieﬂy, a specific 372 bp fragment of *CabZIP25* was obtained by searching the SGN VIGS Tool (https://vigs.solgenomics.net/), which can identify the specificity of the gene fragment and possible off-targets with more precision. The *CabZIP25* cDNA fragment was amplified from the pepper line R9 using gene-specific primers (**Supplementary Table S6**). The PCR product was inserted into the TRV2 vector with Xba I and Kpn I site to generate the *TRV2: CabZIP25*-silencing vector. The empty vector *TRV2: 00* was used as the control and the *TRV2: CaPDS* (phytoene desaturase gene) vector was a symbol of successful gene silencing. The resulting plasmids were separately transformed into *A. tumefaciens* strain GV3101 cells, subsequently, *A. tumefaciens*-mediated virus infection mixing with GV3101 cells harboring TRV1 was injected into the cotyledons of the 2-week-old pepper line R9 to create the VIGS plants. When the photo-bleached phenotype of the pepper plants transformed with *TRV2: CaPDS* appeared in the newly grown leaves, the transcriptions of *CabZIP25* and *CabZIP25* homologs (*CabZIP1* and *CabZIP2*) in pepper plants *TRV2: 00* and *TRV2: CabZIP25* was assessed by quantitative real-time PCR (qRT-PCR) with the specific qRT-PCR primers (**Supplementary Figure S7A, B**, and **Supplementary Table S6**). When the expression of *CabZIP25* in the *CabZIP25-*silenced plants showed approximately 30%-40% of the transcripts level in the *TRV2: 00* control plants and the expression levels of *CabZIP1* and *CabZIP2* showed no differences in all the virus-infected pepper plants; the plants were used for salt stress experiments. The excised leaf discs (0.5 cm in diameter) from the *TRV2: 00* or *TRV2: CabZIP25* plants were treated with different NaCl concentrations (0, 300, and 600 mM) for three days at 25/18°C day/night temperature. The Chlorophyll content of each treatment was measured using the method described previously (Cai et al., 2017). Three independent biological replicates were performed in this experiment. Data were reported as means ± SD.

### Total RNA Extraction and qRT-PCR Analysis

Total RNA was isolated from the leaves of pepper and *Arabidopsis* plants using the RNA extraction kit (Tiangen, Shanghai, China) according to the manufacturer's protocol. The first-strand cDNA was synthesized with PrimeScript™ RT reagent Kit with gDNA Eraser (TaKaRa, Beijing, China). The qRT-PCR analysis was performed using SYBR^®^ Premix Ex Taq™ II (TaKaRa, Beijing, China). The relative transcript levels were calculated according to the 2^−ΔΔCT^ method (Livak and Schmittgen, 2001), in which *CaUBI3* (ubiquitin-conjugating protein-coding gene) and *AtACT2* were used as reference genes for pepper and *Arabidopsis*, respectively. Specific primers are listed in **Supplementary Table S6**.

## Results and Discussion

### Genome-Wide Identification of the Pepper *CabZIP* Gene Family

In order to identify the bZIP TF genes in pepper, both the HMM and BLASTP were performed to search against the Zunla-1 and CM334 genomes to obtain the deduced amino acid sequences. We separately obtained 64 candidate *bZIP* genes in the Zunla-1 genome and 58 genes in CM334 genome. Subsequently, the online programs ExPASy and SMART were used to check the presence of the bZIP motifs with confidence (*E*-value 1). Five candidate genes (Capana12g001248, Capana08g000475, Capana02g001374, Capana10g001986, and Capana04g000551) in the Zunla-1 genome and three candidate genes (CA02g10530, CA00g45190, and CA12g13110) in the CM334 genome were discarded due to an incomplete bZIP domain. After removing the repeated and incomplete gene sequences manually, the remaining 60 *bZIP* gene members were finally confirmed as the pepper *CabZIPs*. All the *CabZIP* sequences came from the Zunla-1 genome except the *CabZIP25*, which was obtained from CM334 genome due to an incomplete bZIP domain in the Zunla-1 genome. For further convenience, unique names were assigned to these pepper *bZIP* genes as *CabZIP1-60* (**Supplementary Table S1**). All details of these *CabZIP* genes are summarized in **Supplementary Tables S1** and **S2**. The numbers of the pepper *bZIP* genes characterized in this study were more than 53 predicted in the plant TF database (PlantTFDB, http://planttfdb.cbi.pku.edu.cn/family.php?fam=bZIP). Compared with other plants, the pepper *CabZIP* family was relatively smaller than most plant *bZIP* families such as *Arabidopsis* (75 members) (Jakoby et al., 2002), rice (89 members) (Nijhawan et al., 2007), apple (114 members) (Li et al., 2016), cucumber (64 members) (Baloglu et al., 2014), and maize (125 members) (Wei et al., 2012), but only larger than castor bean (49 members) (Jin et al., 2014), and grapevine (55 members) (Liu et al., 2014). Previous evidence has shown the number of bZIP members in the monocot plants is relatively larger than that in the dicot plants, this standpoint may be the probable reason for pepper having fewer *bZIP* genes (Wang et al., 2011; Jin et al., 2014; Li et al., 2015). Furthermore, given the enormous pepper genome size (3.48 GB for Zunla-1 and CM3340) (Kim et al., 2014; Qin et al., 2014), we inferred that some additional bZIP proteins may not be detected currently with the originally developed method.

Generally, the proteins encoded by the 60 *CabZIP* genes ranged from 134 (CabZIP52) to 766 amino acids (aa; CabZIP56) in length with an average of 317.5 aa (**Supplementary Table S1**). The average length of the pepper CabZIP proteins was similar to that of *Arabidopsis* (321 aa on average) (Jakoby et al., 2002) and tomato (318 aa on average) (Li et al., 2015) bZIP proteins. Additionally, ExPASy analysis suggested the computation of the theoretical MWs of CabZIP proteins were between 15.48 kDa (CabZIP52) and 82.21 kDa (CabZIP56), and the predicted *P*I values of the CabZIPs ranged from 5.15 (CabZIP22) and 10.12 (CabZIP10). The CabZIPs shared a conserved bZIP domain comprised about 65 aa, in which CabZIP45 owned the shortest bZIP domain with 55 aa, while the bZIP domain of CabZIP7 (83 amino acids) was the longest.

### Phylogenetic Analysis and Classification of the CabZIP Family

**Figure 1:**
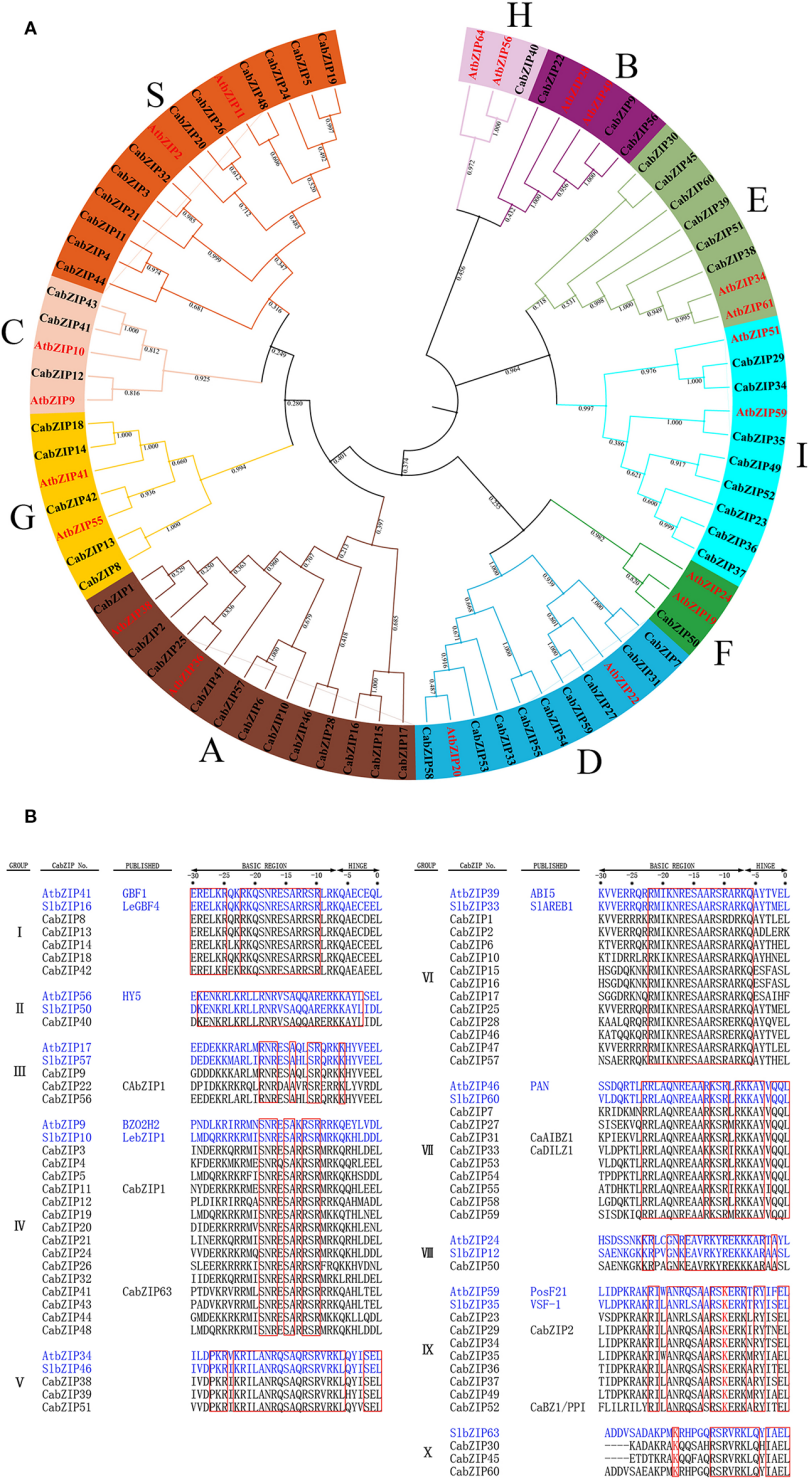
Phylogenetic relationship of pepper and *Arabidopsis* bZIP proteins and alignment of basic and hinge regions of the CabZIP proteins. **(A)** The protein sequences of the bZIPs, 60 from pepper (CabZIPs) and 20 from *Arabidopsis* (AtbZIPs, red color), were aligned by MUSCLE at MEGA-X and the phylogenetic tree was constructed by NJ method. The proteins were clustered into 10 groups, which were assigned a different color. **(B)** Alignment of basic and hinge regions of 60 CabZIP proteins. The CabZIP proteins are classified into 10 groups. Conserved amino acids are boxed in red. Two already characterized plant bZIP proteins in *Arabidopsis* and tomato are shown as references. The first Leu of the Leu zipper is regarded as 0. The different amino acid residue K belonging to group X are colored at -18 positions.

The phylogenetic analysis was performed to investigate the evolutionary relationship of the CabZIP family in pepper. Twenty bZIP protein sequences from the *Arabidopsis* AtbZIP family were added into the tree and used as subgroup markers to classify all the CabZIPs. As shown in **Figure 1A**, all the CabZIP proteins clustered into 10 groups, similar to *Arabidopsis*, designated as clade A to I, and S (Jakoby et al., 2002). Based on the phylogenetic tree, the number of bZIP proteins in each group were 12, 3, 3, 9, 6, 1, 5, 1, 8, and 12 (groups A-I and S), that in *Arabidopsis* were 13, 3, 4, 10, 2, 3, 5, 2, 13 and 17 (group A-I and S), respectively (Jakoby et al., 2002). Except for groups E and I, the number of bZIP proteins in each pepper or *Arabidopsis* group accounted for a similar percentage to their bZIP family members. And it was interesting that the proportion of a new group consisting groups E and I confirmed the classification. Additionally, groups E and I were the adjacent clades in the phylogenetic tree and could be clustered into one group with well-supported bootstrap values (0.964), which were also observed in other studies (Jakoby et al., 2002; Wang et al., 2011; Wang et al., 2015; Zhang et al., 2015; Li et al., 2016). It was speculated that group E had a very close evolutionary relationship with group I, this may lead to the high similarity of gene function in pepper. To further validate the reliability and accuracy of the phylogenetic relationship, the 60 CabZIPs were also categorized into 10 groups (I-X) according to the DNA-binding specificity of basic and hinge regions defined from the first Leu (**Figure 1B**). It was observed that groups I, II, III, VI, VII,VIII, and IX were clustered into the clade G, H, B, A, D, F, and I, respectively (**Figure 1**). Moreover, 15 members in group IV were distributed in clades C (three members) and S (12 members), while groups V and X separately consisted of three members in group E (six members).

Considering the bZIP domain may impact the judgment of the evolutionary relationship, we further compared and analyzed the 60 CabZIP proteins based on their bZIP domains and basic and hinge regions, respectively (**Supplementary Figure S1**). The two phylogenetic trees showed high similarity in clustering the CabZIPs into different clades based on the >50% bootstrap values. Compared with the phylogenetic tree of full CabZIP proteins (**Figure 1A**), the CabZIP22 in group B were clustered into group H, and some neighboring groups were combined into one group in the new two phylogenetic trees. For example, groups E and I in the full proteins tree were clustered into one group, in the bZIP domain and the basic and hinge region trees, further supporting our hypothesis, that there was a very close relationship between groups E and I. The reliability of group classification was further supported by the high similarity grouping characteristics.

To comprehensively study the CabZIPs in terms of phylogenetic relationships among pepper, *Arabidopsis*, and tomato, a total of 201 bZIPs were detected and another phylogenetic tree was constructed (**Supplementary Figure S2**). The inter-species phylogenetic analysis indicated the existence of homologous bZIP TFs in the three plants. In addition, all of the CabZIP and SlbZIP proteins showed a closer evolution relationship than those in AtbZIP proteins because both pepper and tomato belong to the Solanaceae family of plants. For example, certain CabZIP proteins (CabZIP1, CabZIP25, and CabZIP47), belonging to group A, were clustered together with tomato SlbZIP33 (SlAREB1) (Bastias et al., 2014), SlbZIP65 (SlAREB2) (Stankovic et al., 2000) and *Arabidopsis* AtbZIP35 (ABF1), AtbZIP36 (ABF2/AREB1), AtbZIP37 (ABF3), and AtbZIP38 (ABF4/AREB2) (Choi et al., 2000), which are all ABRE-binding proteins known as abscisic acid-responsive elements (ABREs). However, other CabZIP proteins (CabZIP2, CabZIP6, and CabZIP57) in group A were clustered into another clade with several AtbZIP proteins (ABI5, DPBF2, DPBF4/EEL, and AREB3/DPBF3) with ABRE-binding feature (Choi et al., 2000; Uno et al., 2000), the reason was that these bZIP proteins maybe had other additional motifs besides the bZIP domain. These phylogenetic analyses indicated that the evolutionary patterns of bZIP proteins might be similar and the function was probably conserved in different plant species.

### Structure and Conserved Motifs of the CabZIP Proteins

**Figure 2:**
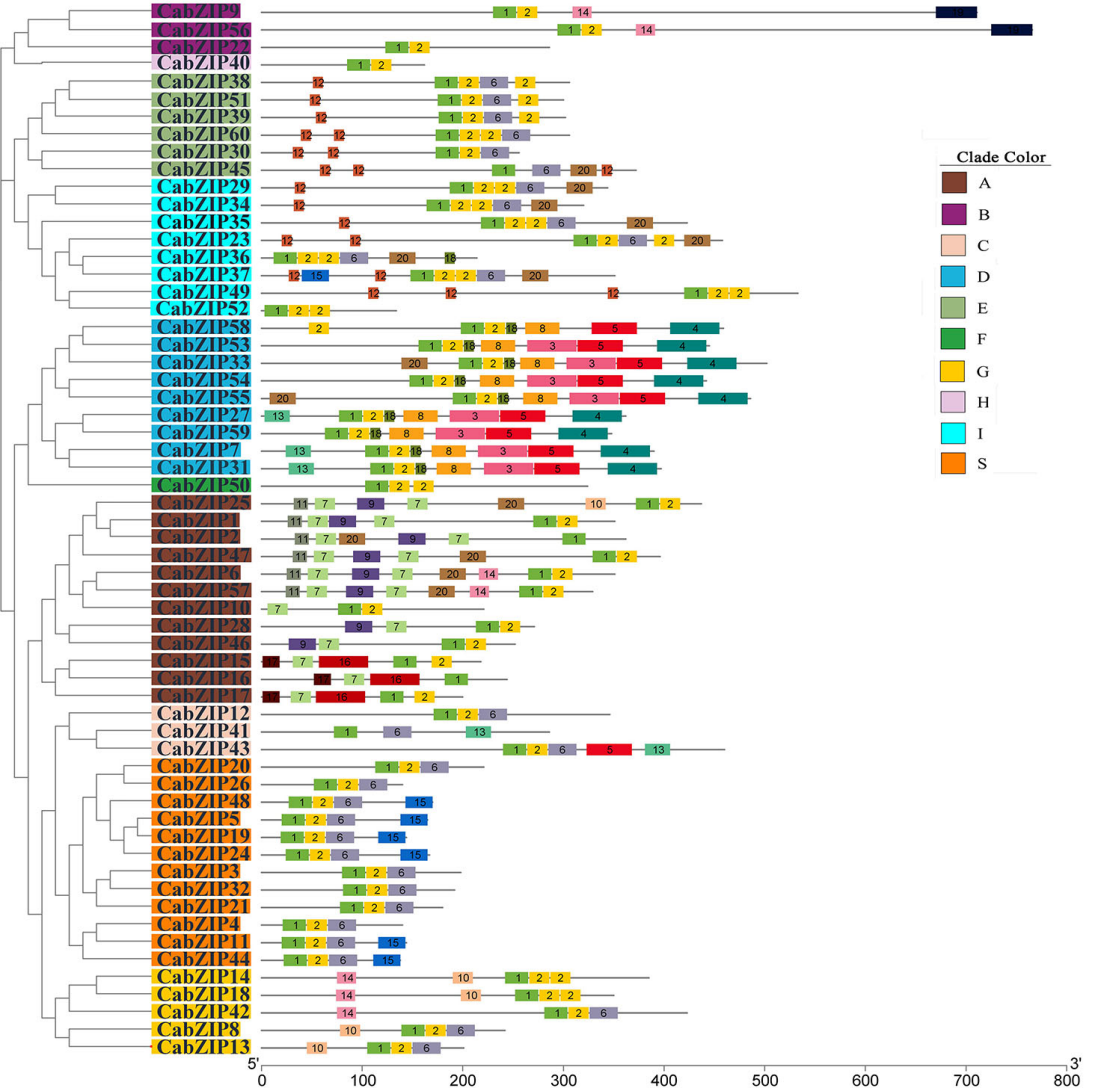
Distribution of the conserved motifs identified in 60 CabZIP proteins. The protein structures of CabZIPs based on the predicted conserved motifs by MEME are given corresponding to the phylogenetic tree of the CabZIP proteins. The phylogenetic relationship and grouping are exactly the same as in **Figure 1**. Each motif is represented by colored box with numbers 1 to 20. The details of conserved motifs are listed in **Supplementary Table S3** and another sketch map of conserved motifs is shown in **Supplementary Table S4**.

The bZIP domain is the key to the function of the bZIP TF family members, and diverse conserved motifs outside of the bZIP domain might indicate the potential functional diversity of *bZIP* genes. In our study, a total of 20 conserved sequences including the bZIP domain were identified in the CabZIP proteins using the MEME tool with low E-value (<E-10). The details of conserved motifs are listed in **Supplementary Table S3** and the distribution of conserved motifs in each CabZIP sequence was shown separately in **Figure 2** and **Supplementary Table S4**. It could be observed that many of the same group members shared the same conserved motifs. Motif 1 (bZIP domain) existed in each sequence, while motif 2 (Leu zipper region) were present in all CabZIP proteins except the CabZIP2, CabZIP16, CabZIP41, and CabZIP45. Based on the sequences alignment of the Leu zipper region, the Leu units of the four CabZIPs without motif 2 were interrupted by two or three other units, and therefore they were not recognized as full Leu zippers by MEME. Additionally, motif 6 was shared by 30 members from groups C, E, G, I, and S; motif 12 was present in groups E and I; motif 10 in groups A and G. However, most of the conserved motifs were present in specific groups. For example, the motif 19 was present in group B exclusively; motifs 11, 16, and 17 only appeared in group A; and group D specifically harbored motifs 3 and 8. Notably, CabZIP40 in group H, CabZIP50 in group F, and CabZIP52 in group I were not detected in any conserved motifs outside the bZIP domain. These observations may signify that the group-specific motifs may determine specific functions of CabZIP proteins in these groups (Jakoby et al., 2002; Nijhawan et al., 2007; Li et al., 2015).

Besides the bZIP domain, we used the motifs known as functional domains to predict the possible biological functions of CabZIP proteins. All nine members of group D contained the TF TGA-related domain (motifs 4, 5, 8, and 18), which are transcriptional activators that bind specifically to the DNA's TGACG sequences and participate in defense against pathogens and development processes (Jakoby et al., 2002; Johnson et al., 2003). These results also suggested that the CabZIP proteins of group D were TGA-subfamily members, and this was supported by the phylogenetic tree of 201 bZIPs, which showed that all members of group D clustered into one group with *Arabidopsis* TGA1/2/3/4/5/6 (**Supplementary Figure S2**). Motifs 7, 9, and 11 represented a potential casein kinase II (CKII) phosphorylation sites (S/TxxD/E), indicated by the motif patterns [TN][LM][ED][DE], TVDE, and T[FL]DE, respectively. These CKII phosphorylation sites in *Arabidopsis* were associated with the ABA signal (Choi et al., 2000). Motif 9 was present in some of the members of group A, a CAMP-response element binding protein-related motif and contained a phosphorylation site (R/KxxS/T) which accounted for RQ[GS]S, for Ca^2+^-dependent protein kinase (Finkelstein and Lynch, 2000; Jakoby et al., 2002).

As reported in previous studies, there were some common motifs outside of the bZIP domain in *Arabidopsis* (Jakoby et al., 2002), tomato (Li et al., 2015), six legumes (Wang et al., 2015), castor bean (Jin et al., 2014), and cucumber (Baloglu et al., 2014). Group A in pepper contained motifs 7, 9, and 11 which were in similar to motifs 1, 2, and 3 of the *Arabidopsis* group A (Jakoby et al., 2002), motifs 7, 8, and 9 of tomato group VI (Li et al., 2015), and motifs 7, 10, and 11 of six legumes group VI (Wang et al., 2015). Motif 20 was commonly shared by pepper, tomato (Li et al., 2015), six legumes (Wang et al., 2015), and castor bean (Jin et al., 2014). Moreover, motif 12 in pepper was the same as motif 1 in tomato (Li et al., 2015), motif 41 in six legumes (Wang et al., 2015), motif 21 in castor bean (Jin et al., 2014), and motif 10 in cucumber (Baloglu et al., 2014), respectively. The further comparison indicated that some common motifs among plant species might be conserved. The *bZIP* genes with the same conserved motifs might imply similar biological gene functions.

### Structure Analysis of *CabZIP* Genes

The intron/exon arrangement of each gene acted as a kind of evolutionary relic to reflect the evolutionary relationship of a gene family (Wei et al., 2012; Wang et al., 2015). To get insights into the structural evolution of the 60 *CabZIP* genes, their intron/exon organization was analyzed (**Supplementary Table S1** and **Supplementary Figure S3**). Since, the most conserved bZIP domains were often considered to be more informative than other regions, we analyzed the exon/intron patterns of the basic and hinge regions for exploring the evolution of *CabZIP* genes based on the distribution, number, and splicing phase of intron/exon. Among the 60 *CabZIP* genes, 13 (21.67%) of the *CabZIP* genes contained no introns, and this phenomenon occurred in groups S and A, all members of group S and *CabZIP16* of group A (**Supplementary Table S1** and **Supplementary Figure S3**). Out of 47 intron-containing *CabZIP* genes, 44 had introns in the basic and hinge regions (**Supplementary Figure S3**). It was also found that different groups had relatively distinct gene structures and the structural patterns of genes within each group were similar (**Supplementary Figure S3**). On the other hand, the number of introns within the ORF varied from 1 to 11 among the intron-containing *CabZIP* genes (**Supplementary Table S1**). Among those having introns, the intron numbers of groups D and G (7-11 and 4-11, respectively) showed the greatest degree of variation (**Supplementary Figure S3**). Meanwhile, the rest of *CabZIP* genes mostly had 1-3 introns except for three members with four to five introns in group E.

Based on the position and presence of introns and splicing phase within the basic and hinge regions of the bZIP domain, *CabZIP* genes were divided into six patterns, which were designated as pattern *a* to *f* (**Supplementary Figures S4** and **S5**). Intriguingly, the pattern of each group was highly conserved, while the intron positions, number, and splicing phases were diverse among each pattern. The intron patterns were similar to those in maize (Wei et al., 2012), tomato (Li et al., 2015), and barley (Pourabed et al., 2015), indicating the gene structures and splicing phases of *bZIP* genes were comparatively conserved within the basic and hinge regions in different plants. Patterns *a*, *b*, *d*, and *e* had a single intron; pattern *c* had two introns and pattern *f* was contained no introns. The pattern *a* had one intron at the -22 position in phase 2 (P2), inserting into the codon which encodes arginine. Pattern *b*, in groups A and G, also had one intron in phase 0 (P0) within the hinge region at the -5 position interrupting the amino acids glutamine and alanine. Two introns, having a P0 splicing phase model, were observed in pattern *c* (the whole group D), and one was in the basic region at the -25 position and the other was at the -5 position interrupting lysine and alanine within the hinge region. Patterns *d* and *e* were exclusive in group E and both of the two patterns inserted in the same position with the P2 splicing phase model. The only difference was the interrupted amino acid residues. Pattern *f* was devoid of any intron in the basic or hinge region and contained 16 *CabZIPs*. It was observed that the splicing phase and position of intron were always conserved, whenever intron was present in the basic and hinge region. And the overall patterns of intron distribution in *CabZIPs* were extremely consistent with those in maize (Wei et al., 2012). It could be inferred that the intron patterns might act as a relatively conservative index during the evolution of *CabZIP* family genes.

### Expression Analysis of *CabZIP* Genes at Different Developmental Stages in Specific Tissues

**Figure 3:**
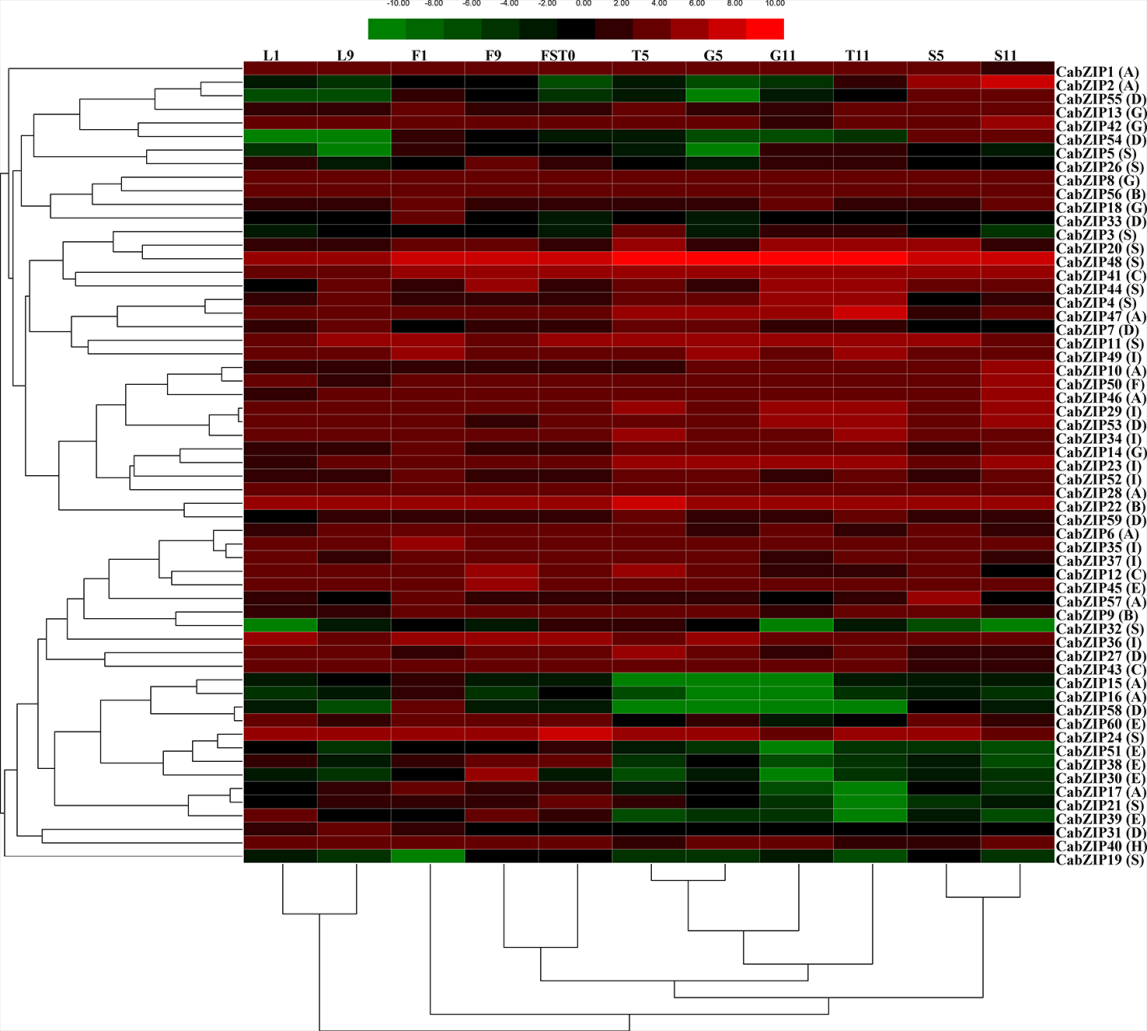
Expression patterns of 59 *CabZIP* genes differentially expressed in six tissues. The image summarized the organ-specific expression profiles of 59 *CabZIPs* in pepper. The expression levels are normalized using log2. The color scale representing expression values is shown at the top. Green indicates low expression values, black indicates intermediate expression values and red indicates high expression values. Genes and the tissues of different developmental stages are clustered according to their expression profiles. Group numbers were labeled after each gene name. The L1 and L9 denote leaf collected at 2 and 60 days after emergence, respectively. The F1 and F9 denotes floral bud collected at smallest and biggest size, respectively. The FST0 denotes fruit collected on 3 days after flowering (DAF). The T5 and T11 denotes placenta collected on 30 and 60 DAF, respectively. The S5 and S11 denotes seed collected on 30 and 60 DAF, respectively. The G5 and G11 denotes pericarp collected on 30 and 60 DAF, respectively.

The bZIP TFs are widely involved in the integration, growth, and development of many plant organs and tissues (Schutze et al., 2008). Increasing shreds of evidences showed the tissue-specific expression patterns of *bZIP* TFs in tomato (Hsieh et al., 2010; Li et al., 2015), cucumber (Baloglu et al., 2014), maize (Wei et al., 2012), and grapevine (Liu et al., 2014). However, the patterns of *bZIP* genes in the regulation of pepper growth and development are still unknown. Thus, to understand the transcription patterns of the *CabZIP* genes in pepper, we performed a hierarchy cluster containing 11 different transcripts, selected from data sets obtained from 57 transcripts, out of 59 *CabZIP* genes (except *CabZIP25*) at different developmental stages in the specific tissues (**Supplementary Table S5**). The expression analysis showed that most of the 59 *CabZIP* genes were expressed in at least one of the six tissues tested and showed an overlapping expression pattern in different tissues (**Figure 3**). For example, the *CaZIP48*, *CaZIP41*, *CaZIP22*, and *CaZIP24* were abundantly expressed among all examined organs. In contrast, the *CaZIP19* and *CaZIP51* were expressed at relatively low levels. Most of the *CabZIP* genes displayed a tissue-specific expression pattern. Two genes (*CabZIP2* and *CabZIP55*) were highly expressed in the seed (S5 and S11), four genes (*CabZIP15*, *CabZIP16*, *CabZIP32*, and *CabZIP58*) were differentially expressed in the floral bud F1 tissue. Interestingly, the homologs of the *CabZIP2* were *AtbZIP67* (*DPBF2*) and *AtbZIP39* (*ABI5*) in *Arabidopsis* (**Supplementary Figure S2**), which were involved in ABA-mediated seed development, germination, and embryo maturation (Bensmihen et al., 2005; Jin et al., 2014; Liu et al., 2014; Wang et al., 2015). The results suggested that *CabZIP2* might have a similar function to the orthologous of the *Arabidopsis* genes, and could be an important TF in ABA-mediated seed development. Compared with other results having three distinct clusters (Nijhawan et al., 2007; Wei et al., 2012), this heat map could be divided into several different clusters according to the hierarchical clustering of 59 *CabZIP* genes in the specific tissues (left side of the heat map). Specifically, *CabZIP1* and *CabZIP19* were clustered into an independent branch. Although a significant relationship between the expression patterns and the gene groups was not found, yet some TFs exhibited a similar expression pattern. For instance, three members (*CabZIP30*, *CabZIP38*, and *CabZIP51*) of group E showed high expression levels in leaf, floral bud, and fruit whereas exhibited low expression in pericarp, placenta, and seed. Based on the dendrogram (hierarchical clustering of six organs at different developmental stages) below the heat map, the heat map could be divided into three clusters. The expression of leaf L1 and L2 were clustered into one branch, suggesting that the expression level in young leaves could be a similar pattern of expression to those of *CabZIP* genes in the old leaves. Moreover, the relatively stable expression patterns of *CabZIP* genes could also be observed in the seeds. Despite of the partial similarity at different developmental stages among the same organs, *CabZIP* TFs had also a different expression pattern in floral bud tissues of pepper. Namely, the *CabZIPs* from opening floral bud F9 shared a higher degree of similarity with fresh fruit FST0 than the unexpanded floral buds F1 in terms of gene expression levels. Therefore, opening floral buds F2 and fresh fruits FST0 were clustered together. These results provided us an important foundation to further understand the functions of *CabZIPs* in pepper's organs.

### Expression of *CabZIP* Genes under Abiotic Stress and Phytohormone Treatments

**Figure 4:**
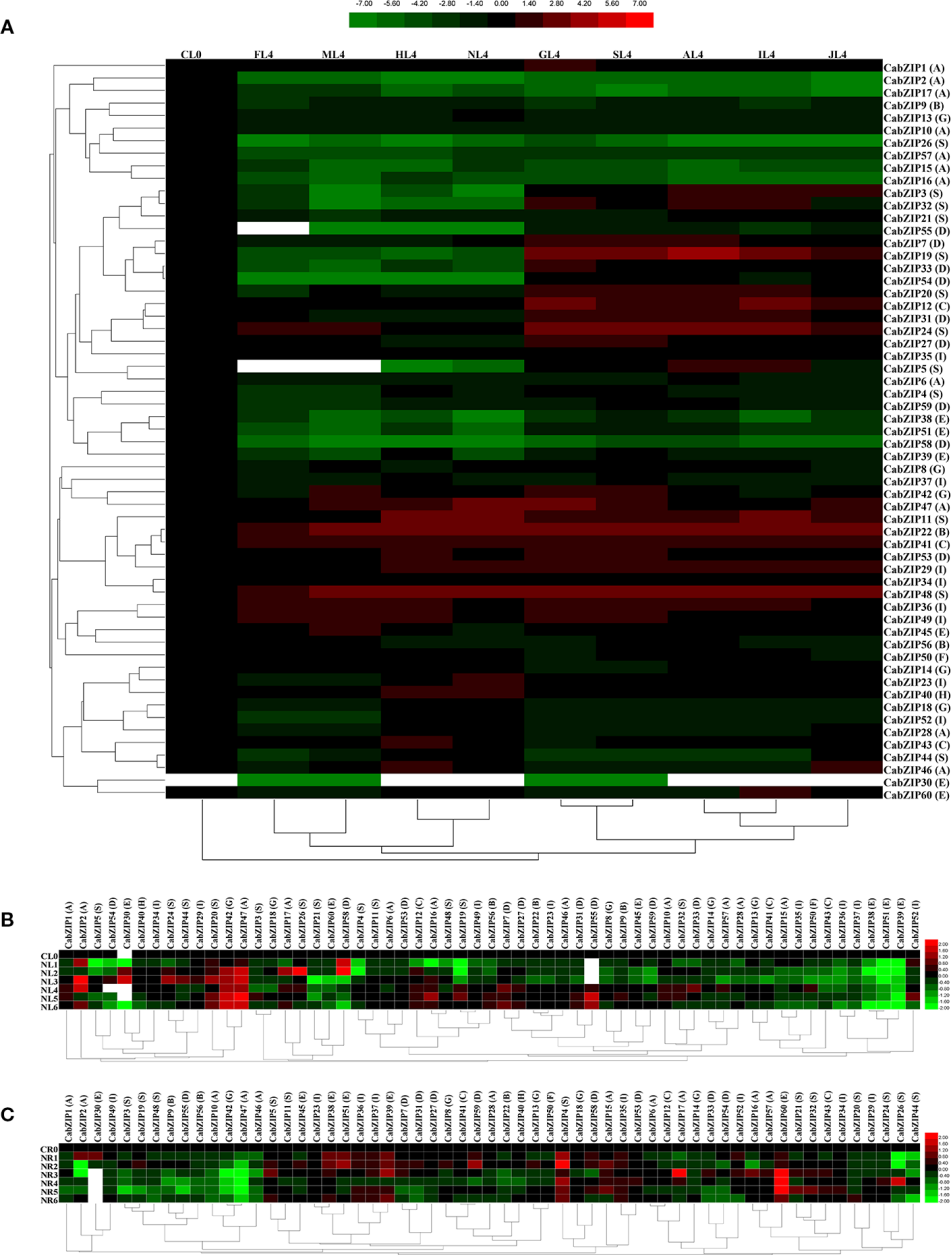
Patterns of 59 *CabZIP* genes differentially expressed during abiotic stress and phytohormone treatments. **(A)** Expression patterns of *CabZIP* genes in leaf at 6 h post treatment. CL0, FL4, ML4, HL4, NL4, GL4, SL4, AL4, IL4, and JL4 represents the control, cold, mannitol, heat, salt, GA3, SA, ABA, IAA, and JA treatment, respectively. **(B**, **C)** Expression patterns of *CabZIP* genes in leaf **(B)** or root **(C)** under salt stress. CL0/CR0, NL1/NR1, NL2/NR2, NL3/NR3, NL4/NR4, NL5/NR5, and NL6/NR6 represents the expression levels of *CabZIP* genes in leaf/root at 0, 0.5, 1, 3, 6, 12, and 24 h post treatment, respectively. Log2 (treated/control) ratio values represents the fold change of the gene expression. The color scale is shown at the top **(A)** or right **(B**, **C)**. Green indicates low expression values; black indicates intermediate expression values and red indicates high expression values. The empty values were indicated as white. Group numbers are labeled after each gene name. See **Supplementary Table S5** for detailed transcriptome data.

Pepper plants are frequently challenged by various environmental stresses, such as salt stress and extreme temperatures. Given the potential roles of *CabZIP* TFs, as a kind of ubiquitous TFs may play important roles in response to multiple stresses by regulating the expression of stress-related genes in pepper. Additionally, phytohormones such as ABA, JA, and IAA act as a wide spectrum of endogenous messengers, which play a crucial role in responses to environmental stresses. Previous studies showed that treatments of plants by exogenous hormones often result in instantaneous and rapid genome-wide transcriptional changes (Seif et al., 2015). Thus, to investigate the possible involvement of CabZIP TFs in the regulation of stress-related genes, the expression patterns of *CabZIP* genes were analyzed under four environmental factors (cold, osmotic, heat, and salt stresses) and five phytohormones (GA3, SA, ABA, IAA, and JA), based on transcriptomic data (**Supplementary Table S5**). The values, which representing a fold change of each *CabZIP* gene compared with the control, are illustrated by a heat map (**Figure 4**). Details normalized as Log2 in treated and control plants.

As shown in **Figure 4A**, the heat map could be divided into three clusters based on the different treatments. Cluster I, where the Log2 (control/control) ratio values were 0. Cluster II and III contained the four abiotic stresses and five phytohormone treatments, respectively. These results showed that the expression pattern of some *CabZIPs* varied between the abiotic stresses and phytohormones, implying their distinctive involvements in the pepper phytohormone signaling pathways and abiotic stimuli. Furthermore, *CabZIP* genes showed a similar expression pattern under two different treatments in the same cluster, such as cold and mannitol stresses or GA3 and SA treatments. These results indicated that the expression patterns of *CabZIPs* could be regulated by different treatments. According to the hierarchical clustering of 59 *CabZIP* genes in response to different treatments, we found that certain down-regulated genes in response to some abiotic stresses were up-regulated by several of phytohormone treatments. For instance, *CabZIP* genes consistent with this expression pattern were mainly the members in group D (*CabZIP7*, *CabZIP33*, *CabZIP31*, and *CabZIP27*) and S (*CabZIP3*, *CabZIP32*, *CabZIP19*, *CabZIP20*, *CabZIP24*, and *CabZIP5*). *CabZIP1*, only up-regulated in the GA3 treatment, was clustered into a single branch and had no significant response to other treatments. Meanwhile, *CabZIP1* was also clustered into an independent branch in the heat map of tissue-specific expression patterns and had high expression levels among all tissues (**Figure 3**). It could be concluded that *CabZIP1* may be specifically involved in the signal pathway of GA and also plays a crucial role in the regulation of the morphology of the plants under the controlling of GA (Fukazawa et al., 2000).

Examination of transcriptome data also indicated that the majority of *CabZIP* genes displayed a differential expression under different treatments (**Figure 4A**). For example, some down-regulated genes induced by JA were up-regulated during the IAA treatment, such as *CabZIP32*, *CabZIP5*, and *CabZIP60*. On the contrary, some genes were up-regulated by the JA treatment but down-regulated by the IAA treatment, such as *CabZIP46* and *CabZIP47* in the group A. The results suggested that some *CabZIP* genes were involved in different biological pathways in response to various phytohormones. Notably, expression of *CabZIP31* in leaves was significantly down-regulated during mannitol, heat, and salt stresses, but was markedly down-regulated by the ABA treatment. This was consistent with previous reports, where *CaAIBZ1* (*CabZIP31*)-silenced pepper and *CaAIBZ1* (*CabZIP31*)-overexpressing *Arabidopsis* exhibited drought-tolerant and sensitive phenotypes, respectively. Whereas, CaAIBZ1 (CabZIP31) degraded by CaASRF1 positively modulates ABA signaling and the drought stress (Joo et al., 2018). By contrast, *CabZIP48*, *CabZIP41*, and *CabZIP22* were significantly up-regulated in leaves during drought, heat, and salt stress conditions, respectively. These results were supported by the research on *CaBZ1* (*CabZIP48*) (Hwang et al., 2005), *CabZIP63* (*CabZIP41*) (Shen et al., 2016), and *CAbZIP1* (*CabZIP22*) (Lee S. C. et al., 2006). The transcript levels of pathogen-induced bZIP TF *CAbZIP1* (*CabZIP22*) (Lee S. C. et al., 2006), *CabZIP2* (*CabZIP29*) (Lim et al., 2015), *CabZIP63* (*CabZIP41*) (Shen et al., 2016), and *CaBZ1/PPI1* (*CabZIP48*) (Lee et al., 2002; Hwang et al., 2005) were up-regulated under stress conditions and phytohormone treatments in our study, implying the involvement of these *CabZIPs* in the signal transduction pathway of biotic stresses.

To further explore the temporal expression patterns of *CabZIP* genes, we analyzed the expression levels of 59 *CabZIP* genes in leaves (**Figure 4B**) and roots (**Figure 4C**) at 0, 0.5, 1, 3, 6, 12, and 24 h post salt stress treatment. We observed the differential expression patterns of *CabZIP* genes in response to salt stress in leaves and roots. The most striking feature was the expression levels of many *CabZIP* genes in pepper leaves and roots were opposite and that same expression pattern could be found in the majority of *CabZIPs* in all groups. For example, *CabZIP42* (group G) and *CabZIP47* (group A) showed highly up-regulated expression in the leaves at a different time points in post salt stress treatment, while the gene expression levels in roots were significantly down-regulated when compared with the control. Moreover, some down-regulated genes induced in leaves were elevated in roots, such as *CabZIP4* (group S), *CabZIP9* (group B), *CabZIP36* (group I), and *CabZIP51* (group E). This observation was consistent with earlier reports where the transcript levels of the apple *MdbZIP60*, *MdbZIP98*, and *MdbZIP109* showed the opposite expression pattern under NaCl treatment in both the leaves and roots (Li et al., 2016). Interestingly, the opposite regulation of *CabZIPs* in both the organs of pepper suggested the involvement of different pathways in salt response. When salinity stress was not only toxic at the cellular level but also impaired by others factors, such as oxidative stress and water deficit conditions. Possibly, some of the *CabZIPs*, which showed the opposite expression trends in the leaves and roots in response to salt stress, could be due to regulating the perception of the ROS as a signal or water deficit. In other biotic and drought stresses, similar results were also reported (Lee S. C. et al., 2006; Baloglu et al., 2014; Li et al., 2016). Additionally, we found that NL4, the expression of *CabZIPs* in leaves at 6 h after treatment (NR4, the expression of *CabZIPs* in roots at 6 h after treatment), was suitable to reflect the overall expression trends of the *CabZIP* genes in response to salt stress. It was understandable that the expression patterns of some *CabZIP* genes (if their functions were involved in response to other stresses and phytohormones treatments) were not detected for the limited samples tested in our study.

### Cloning and Expression Analysis of *CabZIP25* Gene

After the validation and confirmation of 60 *CabZIP* genes (**Supplementary Table S1**), through alignment of the CabZIP25 protein sequence in Zunla-1 and CM334 genomes, there was an incomplete bZIP domain of the CabZIP25 amino acid sequence in Zunla-1 genome (**Supplementary Figures S6A, B**). We investigated the veracity of the *CabZIP25* gene, and a BLASTN was performed with the upstream 1,500 bp promoter regions of the *CabZIP25* gene in both the genomes. The alignment result showed that the *CabZIP25* promoter sequences in both the genomes were almost the same, just the existing insertion-deletion sequence (**Supplementary Figure S6C**). All pieces of evidence indicated that there was a significant genetic variation of the *CabZIP25* gene in pepper line Zunla-1 and CM334. It was speculated that the *CabZIP25* gene played a different role with the presence/absence of the bZIP domain. In order to explore the function of the *CabZIP25* gene, the complete CDS was amplified from the leaf of pepper line R9 using two pairs of *CabZIP25* CDS primers from Zunla-1 and CM334, but only the primers of *CabZIP25* CDS from CM334 gave the results and was further used in the experiment.

Multiple sequence alignments indicated the *CabZIP25* gene was a true bZIP gene (**Supplementary Figure S6B**), and CabZIP25 protein could have the specific and indispensable function as a complete bZIP TF in the pepper. CabZIP25 belonged to group A (**Figure 1**) and the homologous proteins of CabZIP25 in *Arabidopsis* and tomato have been studied such as AtbZIP35 (ABF1), AtbZIP36 (ABF2/AREB1), AtbZIP38 (ABF4/AREB2) (Choi et al., 2000; Jakoby et al., 2002; Hsieh et al., 2010; Orellana et al., 2010; Bastias et al., 2014), and SlbZIP33 (SlAREB1) (Orellana et al., 2010; Bastias et al., 2014) (**Supplementary Figure S2**). Most of the functional information available of AREB/ABF bZIP TFs suggested the roles in ABA or stress signaling and mediating stress-associated gene regulations (Choi et al., 2000; Jakoby et al., 2002; Orellana et al., 2010).

**Figure 5:**
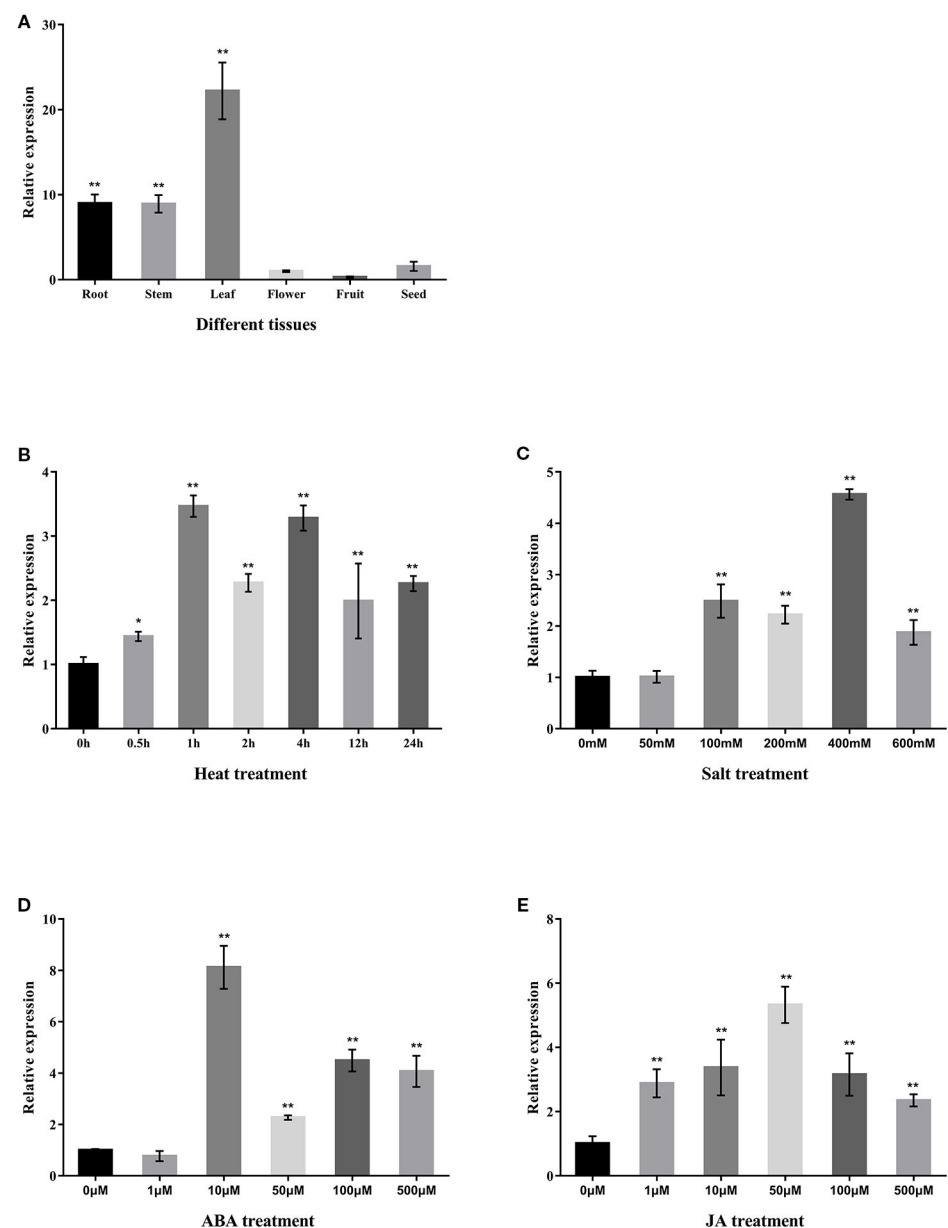
*CabZIP25* expression patterns **(A)** in different pepper tissues, under **(B)** heat stress induction at 45°C, **(C)** NaCl root-immersing, **(D)** ABA spraying, and **(E)** JA spraying. The relative expression levels of *CabZIP25* are normalized to that of *CaUBI3*. Error bars represent SDs for three replicates, and each replicate contains six pepper seedlings. Data are shown as means ± SD. The asterisks on the bars indicate significant differences from the *CabZIP25* expression in flower **(A)** or control treatments **(B**–**E)**. *P < 0.05, **P< 0.01 by Student's *t*-test.

**Figure 6:**
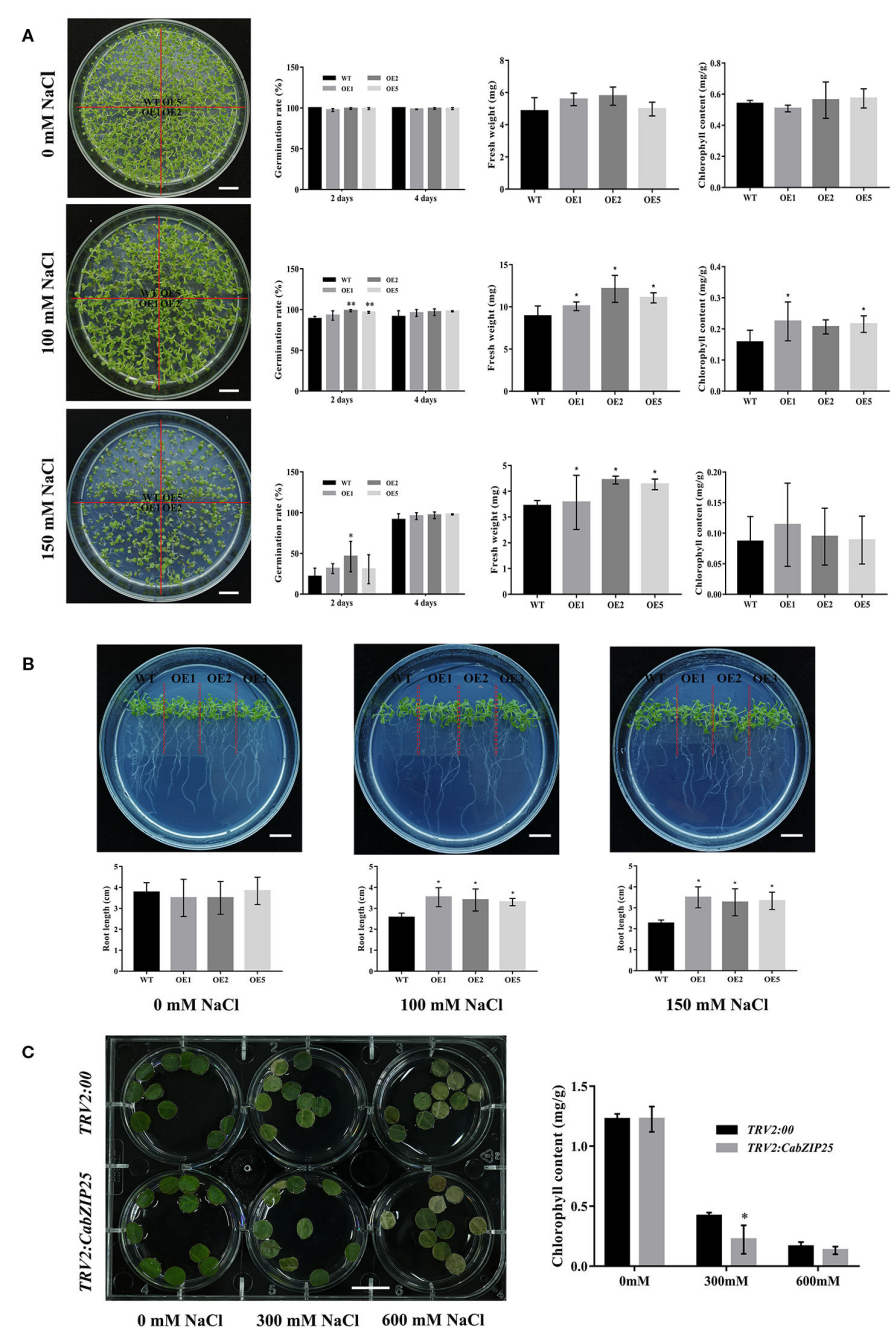
Function analysis of *CabZIP25* under salt stress. **(A)** Phenotypic analysis, seed germination rate, fresh weight, and chlorophyll content of *Arabidopsis* treated with different concentrations of NaCl for 10 days. **(B)** The phenotype and length of *Arabidopsis* roots at 10 days after different concentrations of NaCl. **(C)** The phenotype and chlorophyll content of leaf discs from *TRV2: 00* or *TRV2: CabZIP25* plants. Error bars represent SDs for three replicates. Data are shown as means ± SD. *P < 0.05, **P < 0.01 by Student's *t*-test. Bars = 1 cm.

In order to understand the potential functional role of the *CabZIP25* gene in pepper, we analyzed the expression pattern of the *CabZIP25* in different tissues and response to different treatments in pepper. For the tissue-specific expression of the *CabZIP25* gene, the accumulation of *CabZIP25* mRNA in different pepper tissues was determined by qRT-PCR. Under normal temperature conditions, the *CabZIP25* gene was detected in all tissues and showed significant tissue specificity (**Figure 5A**). As compared to the expression of reproductive tissues, *CabZIP25* was expressed at relatively higher levels in vegetative tissues including roots, stems, and leaves. The *CabZIP25* transcripts were present abundantly in the leaves and the least abundant in fruits. The data suggested that the transcriptional regulation of the *CabZIP25* gene played an active role in the vegetative tissues than reproductive tissues. Whereas, tomato SlbZIP33 (SlAREB1), the homolog protein of CabZIP25, participated in the regulation of the metabolic programming during fruit ripening in tomato (Bastias et al., 2014). The expression of *CabZIP25* in pepper fruits may be affected by temporal and spatial factors of the samples, and the expression analysis might not precisely reflect the potential function of this gene, but whether *CabZIP25* had an important role in other tissues (e.g. during seed maturation) yet to be investigated. In vegetative tissues, ABA and abiotic stresses such as heat, salt, and drought induced gene expression with *cis*-elements including the ABRE element (Jakoby et al., 2002). Thus, we conducted a qRT-PCR analysis of the expression of *CabZIP25* in pepper leaves exposed to heat, NaCl, ABA, and JA for the expression pattern of *CabZIP25* (**Figures 5B–E**). *CabZIP25* in leaves was induced by heat upon initiation of the stressful period (**Figure 5B**). The *CabZIP25* expression was induced to the highest level after 1 h of heat stress and then kept at a high expression level even after 24h of heat stress. After exposure to different concentrations of NaCl, *CabZIP25* expression was strongly enhanced (**Figure 6C**). *CabZIP25* expression was also significantly up-regulated in response to exogenous application of phytohormones such as ABA or JA compared with the control, except for 1µM ABA treatment. Previously, *Arabidopsis AtbZIP36* (*ABF2*/*AREB1*) and *AtbZIP38* (*ABF4*/*AREB2*) genes were also induced by high-salt treatment and exogenous ABA treatment (Choi et al., 2000). In addition, a small numbers of *CabZIPs* were also involved in salt stress and ABA/JA hormones treatments in pepper, such as *CAbZIP1* (*CabZIP2*2) (Lee S. C. et al., 2006), *CaAIBZ1* (*CabZIP31*) (Joo et al., 2018), *CaDILZ1* (*CabZIP33*) (Lim et al., 2018), and *CaBZ1*/*PPI*1 (*CabZIP48*) (Lee et al., 2002; Hwang et al., 2005). Therefore, *CabZIP25* was highly responsive to salt and heat treatments, it could be due to some hormonal signals that participated in enhancing the *CabZIP25* transcription when pepper plants were under the stress environment.

### Performance of the Transgenic *Arabidopsis* and Silenced-Pepper under Salt Stress

Orthologous genes of *CabZIP25* have been reported in other species such as *Arabidopsis* and tomato, and the functional identification of these bZIP TFs revealed that they conferred tolerance to multiple stresses, including salt, heat, and drought stresses (Uno et al., 2000; Jakoby et al., 2002; Fujita et al., 2005; Furihata et al., 2006; Hsieh et al., 2010; Orellana et al., 2010; Bastias et al., 2014). Since the Ca*bZIP25* was strongly up-regulated by salt stress (**Figure 5**), stable or transient expression experiments were used to confirm the function of this TF response to salt tolerance in plants. *CabZIP25* gene was transformed into *Arabidopsis* plants and three homozygous transgenic lines (OE1, OE2, and OE5) were used for further study its function (**Supplementary Figure S7C**).

As shown in the **Figures 6A, B**, all the transgenic lines together with the WT, were grown for 12 days on MS medium supplemented with 0, 100, or 150 mM NaCl, respectively. In the absence of NaCl, *CabZIP25*-OE lines and WT plants showed similar growth including germination rates, fresh weights, chlorophyll contents, and root lengths; while no obvious phenotype changes were observed. However, under salt stress, plant growth was inhibited to various degrees in both the over-expressed lines and WT seedlings with 100 and 150 mM NaCl concentration. After 2 days of treatment with 100 mM NaCl, the germination rates of the OE1, OE2, and OE5 transgenic lines were 22.43%, 20.65%, and 21.54%, respectively, whereas the germination rate of the WT was only 15.76% (**Figure 6A**). The similar results were also obtained for plants cultivated on medium supplemented with 150 mM NaCl after 2 days. Higher germination rates were recorded in the medium containing 150 mM NaCl than that in the WT, however, only the germination rate of the OE2 line was significantly different from that of control (**Figure 6A**). After 4 days, the germination rates of different *Arabidopsis* lines were practical unanimity on all medium. We found that the over-expression of the *CabZIP25* gene could increase the germination rates of transgenic lines under 100 mM salt treatments; at higher concentration, the stress response was there but was not significant. Additionally, both root lengths (**Figure 6B**) and fresh weights (**Figure 6A**) were significantly higher in three transgenic lines than that in WT seedlings under 100 mM and 150 mM salt treatments, while there was no difference between the transgenic and WT seedlings under normal conditions. It was interesting that the fresh weight of *Arabidopsis* grown at 100 mM NaCl was higher than those of control (**Figure 6A**). But many WT and overexpression plants showed a dark color of leaves and abnormal phenotype of seedlings at 100 mM NaCl treatment, indicating that the natural growth of plants had been affected by salt. The fresh weights of transgenic lines were still significantly higher than that in WT seedlings. The possible reason might be that the equilibrium of water and ions in plant cells was broken under salt treatment and the hyperosmotic stress and ion imbalance caused by salt stress led to abnormal growth and development of plants. But the factual mechanism of this phenomenon remained to be further explored. Previous studies have shown that over-expression of *SlAREB1* (*SlbZIP33*) resulted in a noticeably improved tolerance to salt (Hsieh et al., 2010; Orellana et al., 2010), and pepper *CAbZIP1* (*CabZIP22*) transgenic *Arabidopsis* plants displayed enhanced tolerance to salt stresses (Lee S. C. et al., 2006). In our study, CabZIP25 also showed a vital role in regulating seedlings growth in response to salt stress. Hence, to explore the latent reason affecting the seedlings growth situation, we monitored the chlorophyll contents of 12 days seedlings under different salt treatments (**Figure 6A**). There was no difference in the chlorophyll contents between the transgenic and WT *Arabidopsis* seedlings under normal conditions, however, *CabZIP25*-OE lines displayed higher chlorophyll contents than WT on medium supplemented with 100 mM NaCl. Similarly in tomato, total chlorophyll content in the salt-stressed plants was much higher than that of *SlAREB1* (*SlbZIP33*)-down-regulating plants, while much higher chlorophyll content was detected in the *SlAREB1* (*SlbZIP33*)-over-expressed plants than that of the WT control (Orellana et al., 2010). The Chlorophyll content could reflect the level of damage during the stress assays. Surprisingly, the chlorophyll contents of *CabZIP25*-OE lines did not differ significantly from that of WT plants under 150 mM NaCl treatment in our study.

To further validate the results, *CabZIP25* was silenced in the pepper line R9. The appearance of photo-bleached leaves of *TRV2: CaPDS* pepper plants as a positive control, approximately 4 weeks after the infiltration of the media, showed a successful silencing through VIGS. Although, no visible difference was observed the phenotype in *TRV2: 00* (control) and *TRV2: CabZIP25* silenced pepper plants (**Supplementary Figure S7A**). Then qRT-PCR analysis was performed to measure the silencing efficiency of the silenced gene with the control. As compared to the *TRV2: 00* plants, the expression level of *CabZIP25* in the silenced pepper plants was 64% lowered than the control plants (**Supplementary Figure S7B**). Additionally, the homologous genes of *CabZIP25* (*CabZIP1* and *CabZIP2*) were almost equally expressed in all the virus-infected pepper plants (**Supplementary Figure S7B**). Subsequently, the excised leaf discs from the *TRV2: 00* and *TRV2: CabZIP25* plants were exposed to 0, 300, or 600 mM NaCl solution for three days, respectively (**Figure 6C**). The results showed that the leaf discs of *TRV2: 00* or *TRV2: CabZIP25* plants under salt treatments exhibited lower chlorophyll contents, as compared to the control treatment. Under 300 mM NaCl treatment, the chlorophyll contents decreased from 1.23 to 0.22 mg/g in the leaf discs of plants with *TRV2: CabZIP25*; whereas the chlorophyll contents in the leaf discs of the *TRV2: 00* plants decreased from 1.22 to 0.42 mg/g. After salt treatment, the chlorophyll contents were significantly lower in the leaf discs of plants with *TRV2: CabZIP25* than those with the *TRV2: 00*. Moreover, with 600 mM NaCl stress, the chlorophyll content declined continuously in the leaf discs of plants transformed with both *TRV2: 00* and *TRV2: CabZIP25*, but the average value in the latter (0.13) was not significantly lower than the former (0.16). The results showed that the salt stress decreased the chlorophyll contents of the pepper plants and the chlorophyll contents in *CabZIP25*-silenced plants were lower than that in control plants under salt stress. Previously, wheat *Wabi5*, as a counterpart of AREB/ABF, also improved the abiotic stress tolerance against salt, low temperature, and osmotic stresses in the transgenic tobacco plants (Kobayashi et al., 2008). Our result showed that a lower level of *CabZIP25* expression resulted in reduced tolerance to the injury from salt stress in pepper (**Figures 6A, B**). Taken together, the *CabZIP25* enhanced salt tolerance by inhibiting the breakdown of chlorophyll at a lower concentration of salt in plants through overexpression and silencing of *CabZIP25* in the *Arabidopsis* and pepper, respectively. However, no significant improved tolerance to the high-concentration of salt, need further investigation to verify.

## Conclusion

bZIP TF families have been identified in different plants and bZIP TFs play a crucial role in various developmental processes and biotic/abiotic stress responses. However, only a few pepper CabZIPs have been studied so far, a systematic study on the biological functions on the pepper *CabZIP* family is lacking. In the present study, a genome-wide analysis of the *CabZIP* gene family was performed in pepper and a total of 60 *CabZIPs* were obtained from the two pepper genomes. We studied the phylogenetic relationship of *CabZIP* genes, the conserved amino acid residues within the bZIP domain, conserved motifs, intron phases within the basic and hinge regions. Expression analyses based on the transcriptomic data for the 59 *CabZIP* genes were performed under different tissues and in response to different abiotic stresses and phytohormones. In addition, the expression of *CabZIP25* was also measured through qRT-PCR under abiotic stresses (salt and heat) and phytohormones (ABA and JA). Based on the expression results, we further characterized the function of *CabZIP25* through over-expression in *Arabidopsis* and silencing through VIGS in pepper. The results showed that *CabZIP25* enhanced the salt tolerance by maintaining the chlorophyll stabilization in plants. Therefore, our result will provide a better reference for the functional characterization of *CabZIP* family members in plants development and response to environmental stimuli.

## Data Availability Statement

All datasets generated and analyzed for this study are included in the article/**Supplementary Material**.

## Author Contributions

W-XG, XM, and Z-HG contributed to the experimental design. W-XG and XM performed bioinformatics analysis. Y-MQ, B-HS, Q-HL, K-KL, and A-MW performed plant transformation work. W-XG and Z-HG wrote the article. K-KL helped to analyze the data. SH and K-KL reviewed and edited the manuscript. All authors have read and approved the final manuscript.

## Funding

This work was supported through the funding from National Key R&D Program of China (No. 2016YFD0101900), the National Natural Science Foundation of China (No. U1603102 and No. 31272163) and Key Scientific Research Project for Higher Education Institutes of Henan (No. 14A210019). 

## Conflict of Interest

The authors declare that the research was conducted in the absence of any commercial or financial relationships that could be construed as a potential conflict of interest.
