# Supplementary material for: Characterization of the bZIP Transcription Factor Family in Pepper (Capsicum annuum L.): CabZIP25 Positively Modulates the Salt Tolerance
Source: Front Plant Sci. 2020 Feb 26;11:139. doi: 10.3389/fpls.2020.00139 (PMC7054902; doi:10.3389/fpls.2020.00139)
Supplement: Supplementary file 1 [file DataSheet_1.docx]

Supplementary Material

**Supplementary Figure S1.** Phylogenetic analysis among the CabZIP proteins based on bZIP domain basic and hinge regions. The amino acids sequences from the 60 CabZIP proteins are aligned by MUSCLE at MEGA-X and phylogenetic tree is constructed by NJ method. Bootstrap values from 1000 replicates are indicated at each node. The amino acids sequences are clustered into 10 groups, which are assigned a different color.

**Supplementary Figure S2.** Phylogenetic relationship among the pepper, Arabidopsis and tomato bZIP proteins. All bZIP protein sequences are aligned by MUSCLE at MEGA-X and phylogenetic tree is constructed by NJ method. Bootstrap values from 1000 replicates are indicated at each node. Protein names of already characterized bZIP proteins have been indicated.

**Supplementary Figure S3.** The map of intron-exon arrangement of *CabZIP* genes. The black numbers denote the positions of intron. P0 represent the intron splicing site between codons, P2 means the intron splicing site locating after the second nucleotide in one codon.

**Supplementary Figure S4.** Intron patterns within the basic and hinge regions of the bZIP domains in CabZIP proteins. *CabZIP* genes are divided into six intron patterns (*a-f*). An example of a sequence in the basic and hinge regions is shown at the top. The black bars represent the sequences in different intron patterns and the black vertical lines denote the positions of intron. phase 0 (P0) represents the intron splicing site between codons, phase 2 (P2) means the intron splicing site locating after the second nucleotide in one codon.

**Supplementary Figure S5.** The details of intron patterns within the basic and hinge regions of the bZIP domains in CabZIP proteins. The red vertical lines denote the positions of intron. *CabZIP* genes are divided into six intron patterns (*a-f*) followed by the number of genes.

**Supplementary Figure S6.** Alignments for the (A) protein, (B) nucleotide, and (C) promoter sequences of *CabZIP25* gene from CM334 (CA04g18620), Zhunla-1 (Capana04g000551), and line R9 (CabZIP25-R9).

**Supplementary Figure S7.** Confirmation of *CabZIP25*-silenced pepper seedlings and *CabZIP25*-overexpressing Arabidopsis lines. (A) Phenotypes of the pepper seedlings 30 d after inoculation. *TRV2: PDS*, seedlings with the *TRV2: PDS* vector (PDS, phytoene desaturase gene for chlorophyll synthesis); *TRV2: 00*, control seedlings with the empty TRV2 vector; *TRV2: CabZIP25*, *CabZIP25*-silenced seedlings. (B) The alignment of the *CabZIP25*-VIGS sequence with its [homologous](javascript:;) [sequence](javascript:;) (*CabZIP1* and *CabZIP2*) and the efficiency of gene expression silencing in *CabZIP25*-silenced pepper. qRT-PCR was performed to analyze the expression with the specific qRT-PCR primer (Supplementary Table S6). The *CaUBI3* gene was used as a reference. ∗ indicates significant difference at the 0.05 levels by Student's *t*-test. (C) Determination of gene expression levels in *CabZIP25*-overexpressing Arabidopsis lines. WT, wide type Arabidopsis line; OE, Arabidopsis transgenic lines with *CabZIP25* gene. qRT-PCR primers of *CabZIP25* were used for amplification and *AtACT2* gene was used as the internal control. Primers are listed in Supplementary Table S6.

**Supplementary Table S1.** Information on the pepper CabZIP family.

**Supplementary Table S2.** Protein sequences of CabZIP transcription factors.

**Supplementary Table S3.** The conserved motifs identified from 60 CabZIP proteins.

**Supplementary Table S4.** The sketch map of conserved motifs of 60 CabZIP proteins.

**Supplementary Table S5.** Transcriptomic data of 59 *CabZIP* genes.

**Supplementary Table S6.** Primers used in this study.
