# Supplementary figures and images for: Characterization of the bZIP Transcription Factor Family in Pepper (Capsicum annuum L.): CabZIP25 Positively Modulates the Salt Tolerance
Source: Front Plant Sci. 2020 Feb 26;11:139. doi: 10.3389/fpls.2020.00139 (PMC7054902; doi:10.3389/fpls.2020.00139)

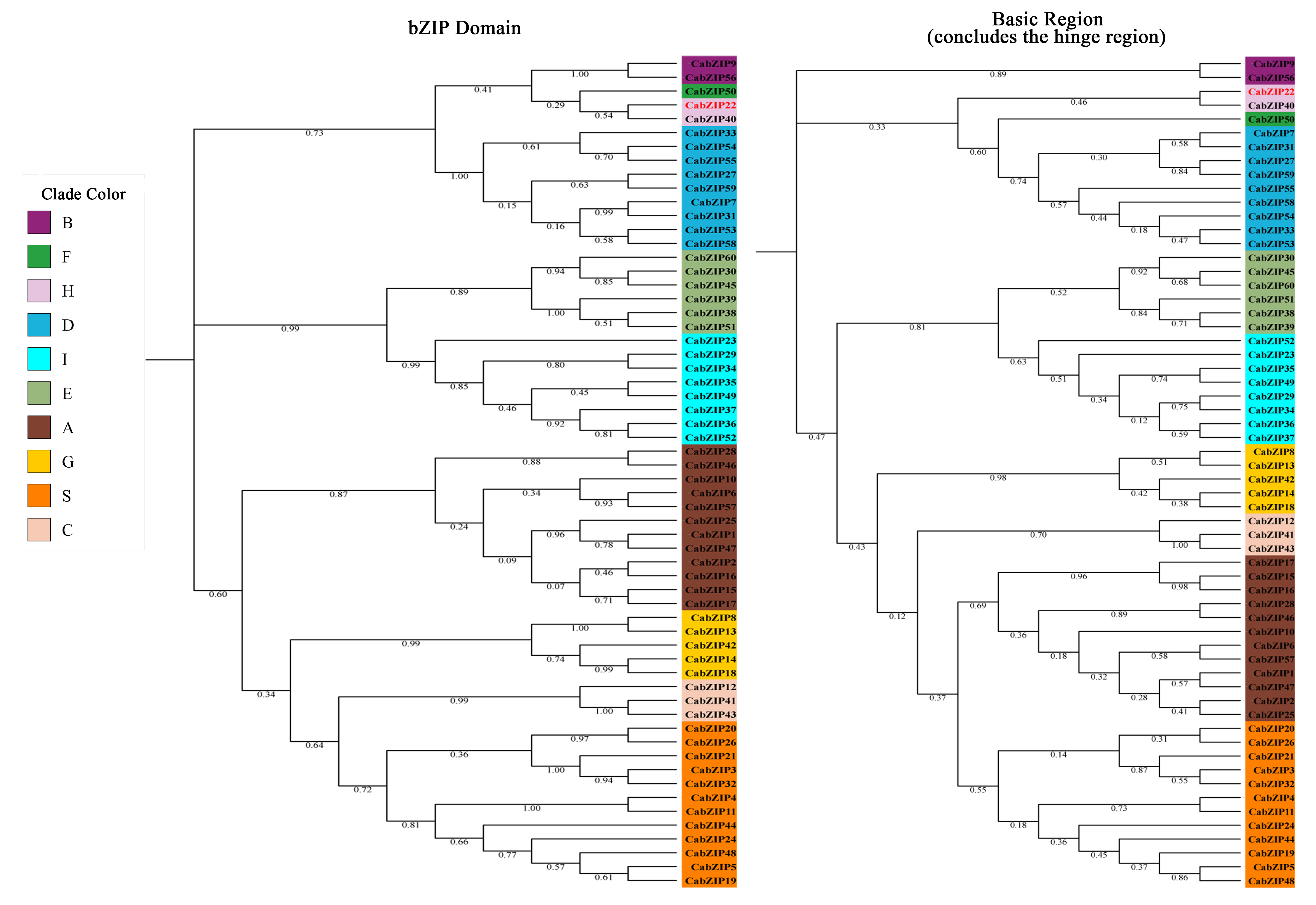

Supplement: Supplementary file 2 [file Image_1.jpeg]

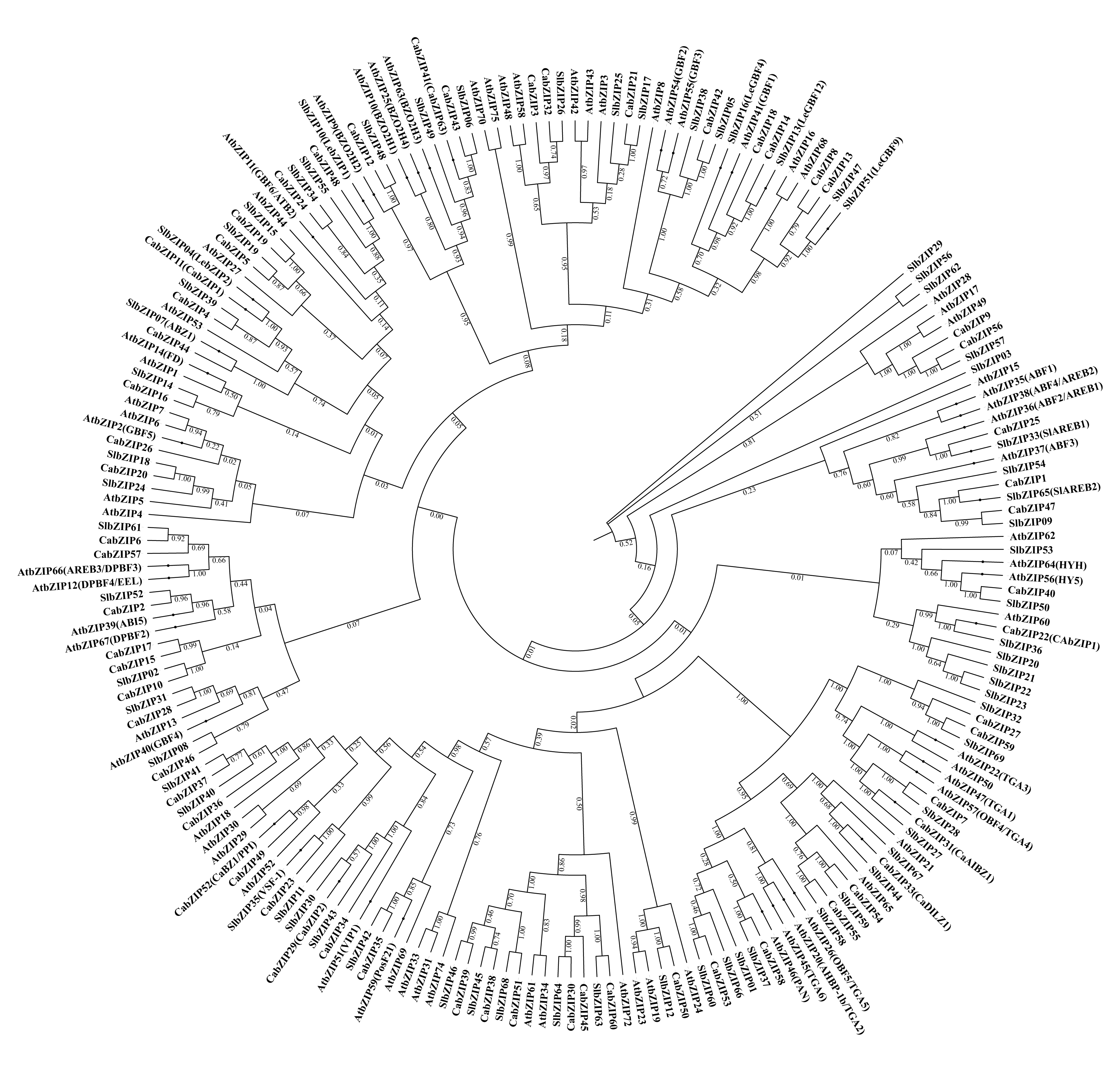

Supplement: Supplementary file 3 [file Image_2.jpeg]

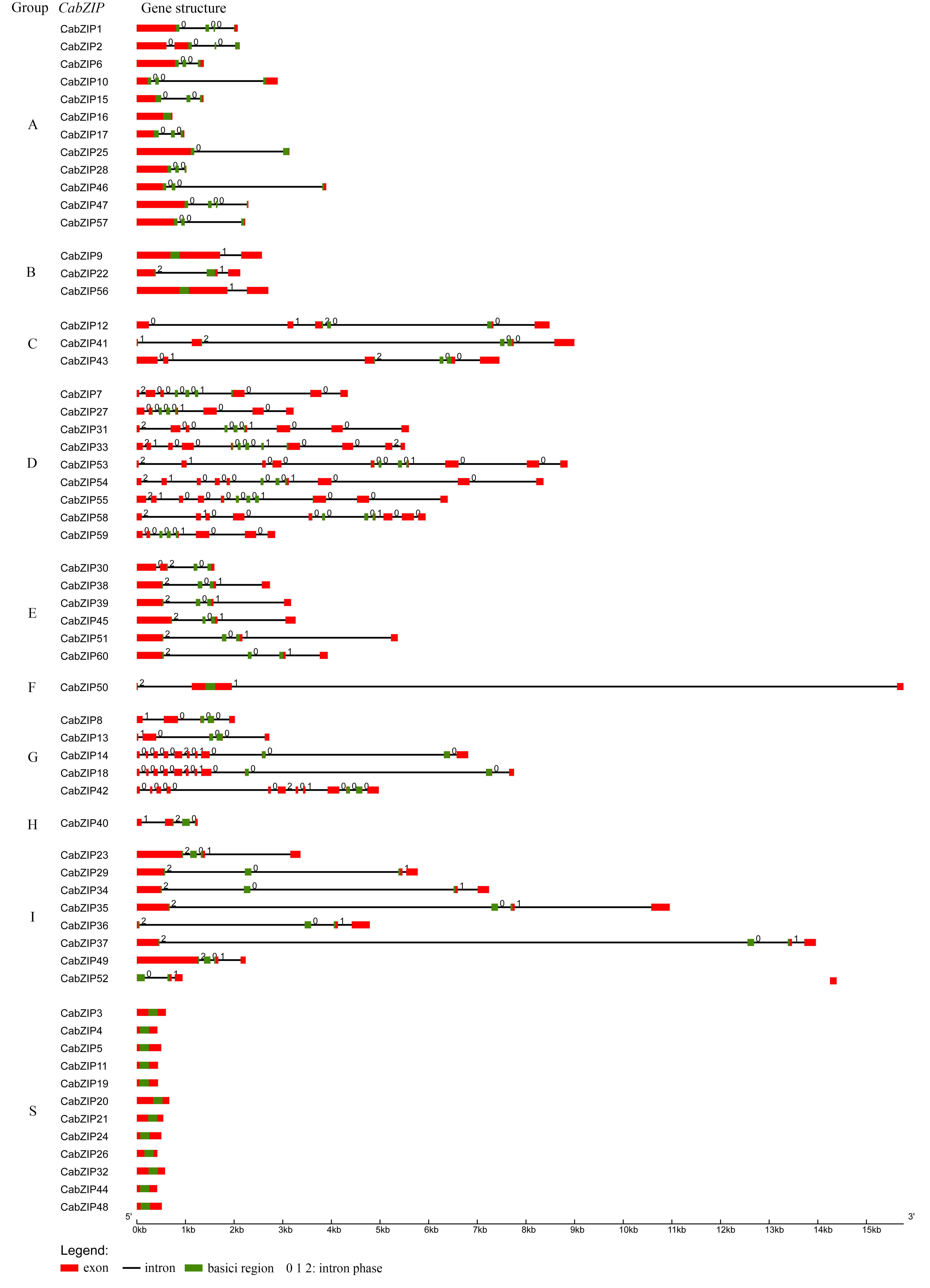

Supplement: Supplementary file 4 [file Image_3.jpeg]

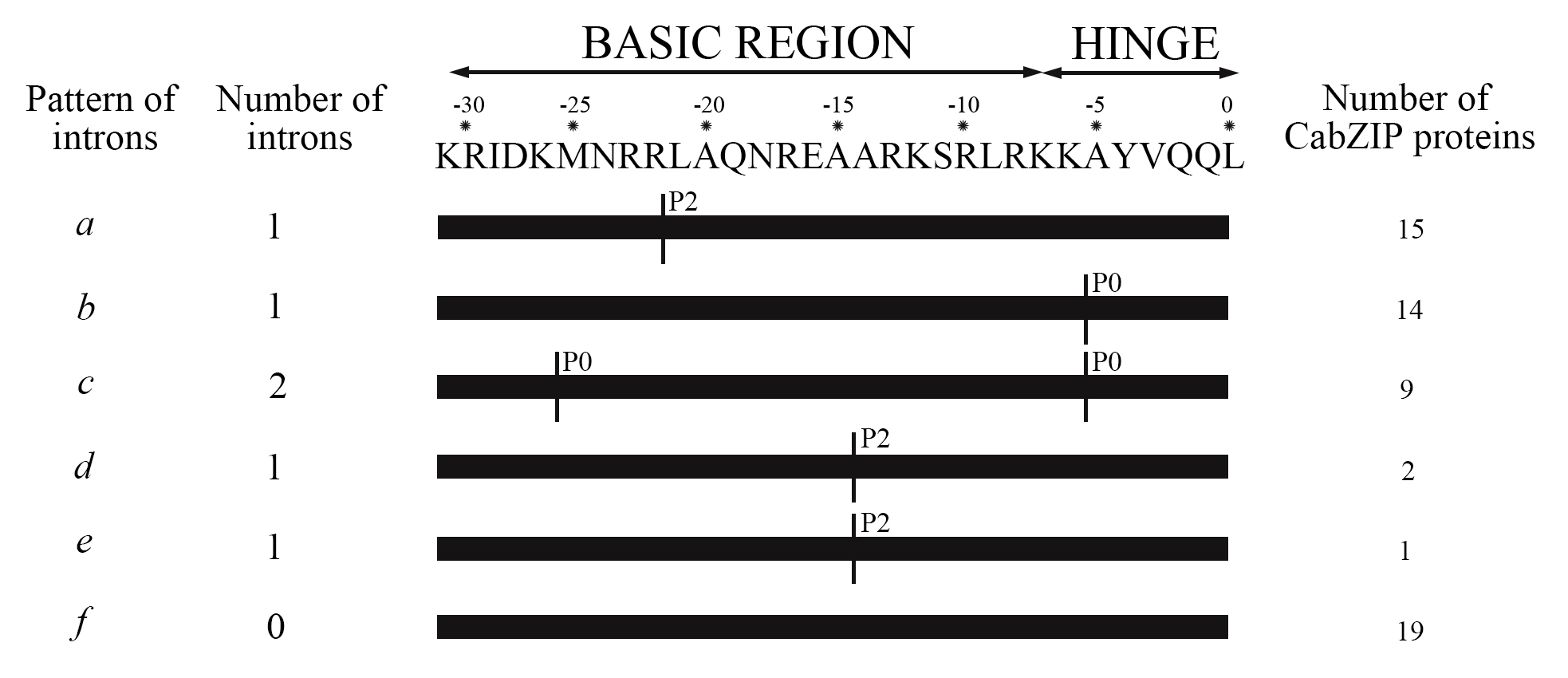

Supplement: Supplementary file 5 [file Image_4.jpeg]

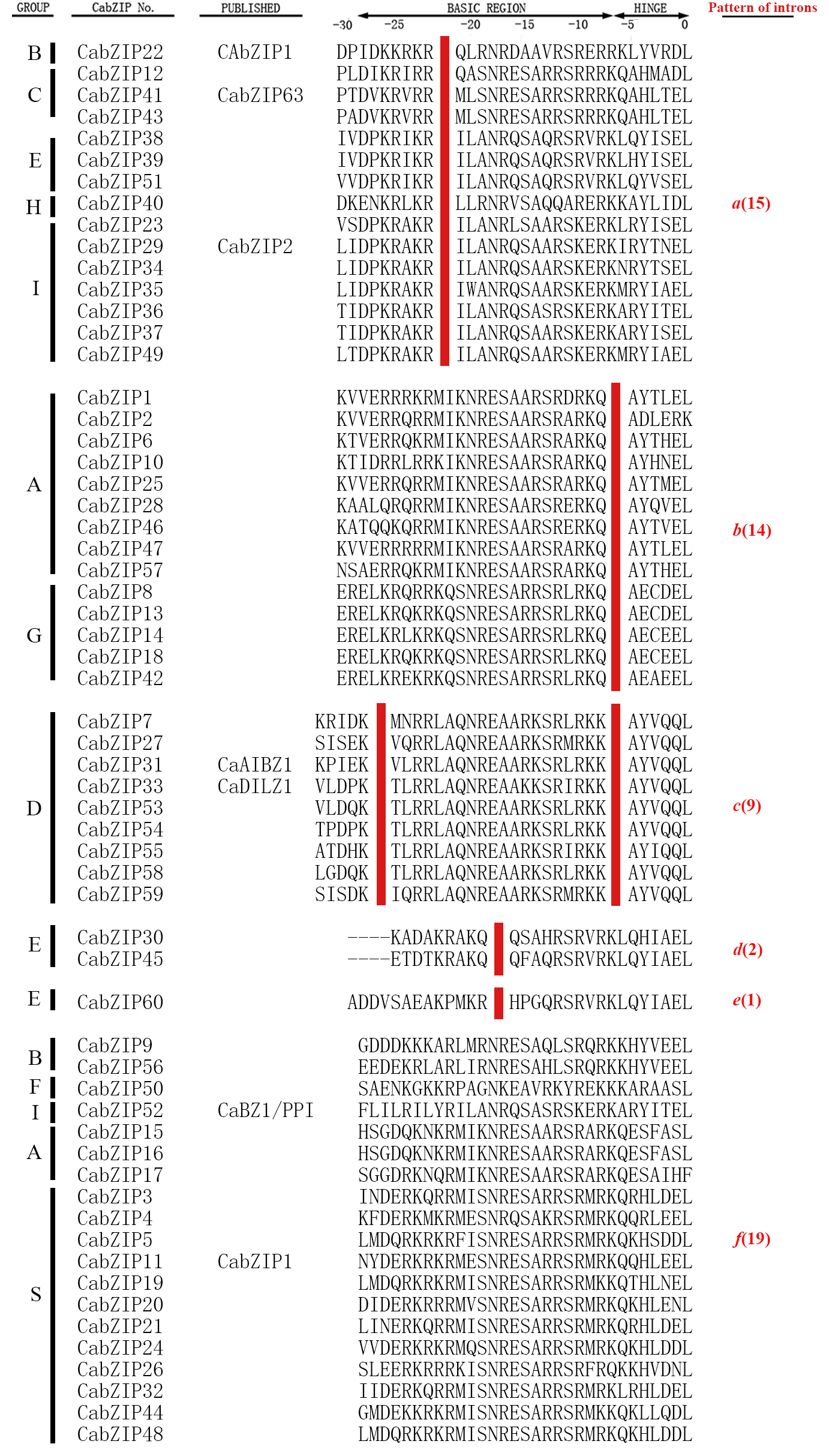

Supplement: Supplementary file 6 [file Image_5.jpeg]

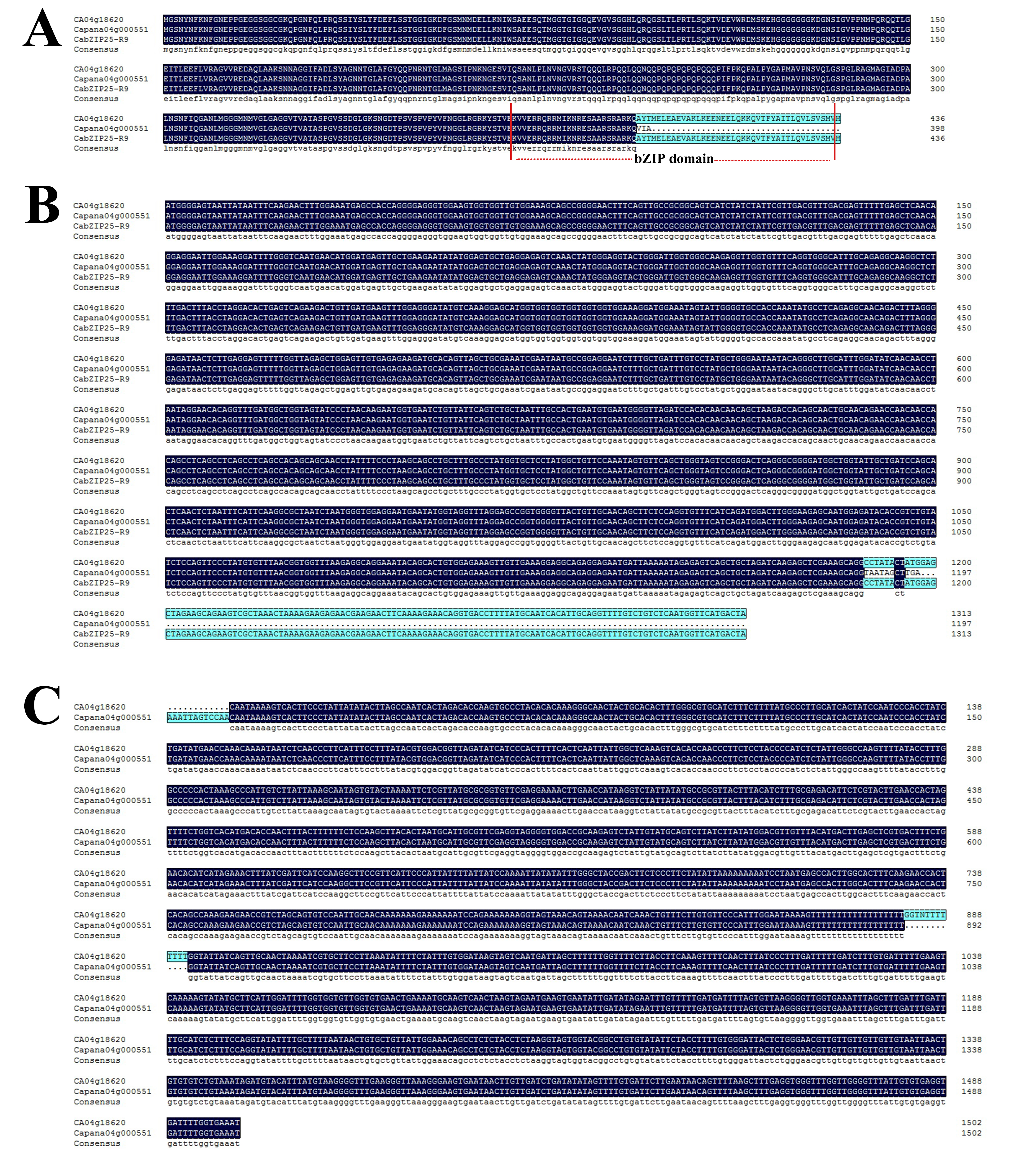

Supplement: Supplementary file 7 [file Image_6.jpeg]

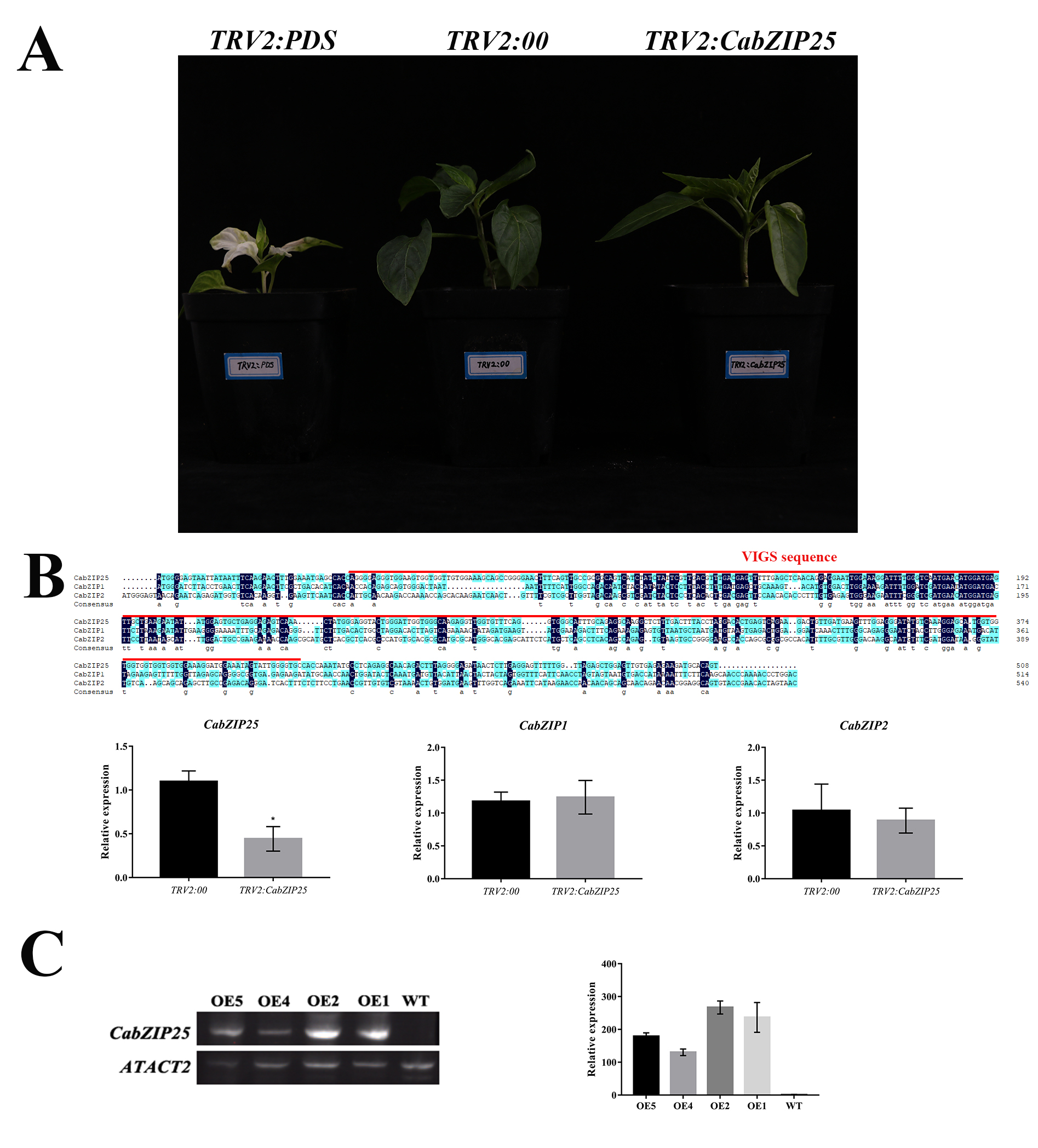

Supplement: Supplementary file 8 [file Image_7.jpeg]
