# Supplementary material for: Characterization of the bZIP Transcription Factor Family in Pepper (Capsicum annuum L.): CabZIP25 Positively Modulates the Salt Tolerance
Source: Front Plant Sci. 2020 Feb 26;11:139. doi: 10.3389/fpls.2020.00139 (PMC7054902; doi:10.3389/fpls.2020.00139)
Supplement: Supplementary file 14 [file Table_6.doc]

**Supplementary Table S6 Primers used in this study**

| Primer Name | **Forward (5'–3')** | Reverse (**5'–3')** |
| --- | --- | --- |
| **For cloning*** |  |  |
| *CabZIP25-1*  (CM334) | GCTCTAGATGGTGAAATGGGGAGTAA | GGGGTACCGTCATGAACCATTGAGACAG |
| *CabZIP25-2*  (Zunla-1) | GCTCTAGATGGTGAAATGGGGAGTAA | GGGGTACCAGCTATTACCTGCTTTCGA |
| *CabZIP25*  (VIGS) | GCTCTAGACAGGGGAGGGTGGAAG | GGGGTACCGCACCCCAATACTATTTCC |
| **For qRT-PCR** |  |  |
| *CaUBI3* | TGTCCATCTGCTCTCTGTTG | CACCCCAAGCACAATAAGAC |
| *AtACT2*  (AT3G18780) | ATGAAGCACAATCCAAGAGAGGTATTCTT | GAGCTTCTCCTTGATGTCTCTTACAATTTC |
| *CabZIP25* | AAGAACTTTGGAAATGAGCC | CAACGAATAGATAGATGACTGC |
| *CabZIP1* | TTTCTTCAAGCAACCCAA | TGCTCATAACACCACCCT |
| *CabZIP2* | CATTTGGACTGCCGAAGA | GCTGCTTGACAATACCCTTA |

*Sequences of the enzyme cutting sites are underlined
